# Representation of Attended Versus Remembered Locations in Prefrontal Cortex

**DOI:** 10.1371/journal.pbio.0020365

**Published:** 2004-10-26

**Authors:** Mikhail A Lebedev, Adam Messinger, Jerald D Kralik, Steven P Wise

**Affiliations:** **1**Laboratory of Systems Neuroscience, National Institute of Mental HealthBethesda, MarylandUnited States of America

## Abstract

A great deal of research on the prefrontal cortex (PF), especially in nonhuman primates, has focused on the theory that it functions predominantly in the maintenance of short-term memories, and neurophysiologists have often interpreted PF's delay-period activity in the context of this theory. Neuroimaging results, however, suggest that PF's function extends beyond the maintenance of memories to include aspects of attention, such as the monitoring and selection of information. To explore alternative interpretations of PF's delay-period activity, we investigated the discharge rates of single PF neurons as monkeys attended to a stimulus marking one location while remembering a different, unmarked location. Both locations served as potential targets of a saccadic eye movement. Although the task made intensive demands on short-term memory, the largest proportion of PF neurons represented attended locations, not remembered ones. The present findings show that short-term memory functions cannot account for all, or even most, delay-period activity in the part of PF explored. Instead, PF's delay-period activity probably contributes more to the process of attentional selection.

## Abstract 2

Persistent activity of neurons in an area of the frontal lobe - the prefrontal cortex - is often proposed to underlie short- term memory. Mikhail Lebedev and colleagues provide an alternative explanation

## Introduction

Jacobsen (1935, 1936) first discovered that damage to the primate prefrontal cortex (PF) appeared to cause a short-term memory deficit. In his experiments, monkeys and chimpanzees with bilateral damage to PF failed to retrieve food from one of two opaque cups when the food had been out of sight for even a few seconds. Intact animals could find the food 5 min or more after they had last seen it. Pribram et al. (1952) later identified the part of PF responsible for this deficit as area 46, also known as the dorsolateral prefrontal cortex (PFdl). More recently, temporary inactivations of portions of PFdl caused what appeared to be a short-term memory loss in localized regions of space (Funahashi et al. 1993a).

Once the concept of working memory (Baddeley 1986) became established in contemporary neuroscience (see Postle et al. 2003), these neuropsychological findings contributed to the theory that PF functions in working memory (Goldman-Rakic 1987) and, in some extreme formulations, only in working memory. In the 1990s this theory developed a wide following, and the idea that PFdl functions in spatial working memory, with other parts of PF functioning in different kinds of working memory, became the predominant theory of PF function, especially for nonhuman primates. As important, the concept of working memory used by proponents of this theory focused mostly on the short-term maintenance of information, and rather less on the manipulation or monitoring of such information or on the use of that information for decisions. Accordingly, we refer to the former aspect of working memory as *maintenance memory* to distinguish it from the broader concept and do not use the phrase *working memory* elsewhere in this report. Note, however, that when we use the phrase maintenance memory, many authorities would use “working memory” instead.

Consistent with the idea that PF functions predominantly in maintenance memory, delay-period activity in PF has often been interpreted as a memory trace (e.g., Funahashi et al. 1989; Romo et al. 1999; Constantinidis et al. 2001). The phrase *delay-period activity* applies to neuronal activity that follows the transient presentation of an instruction cue and persists until a subsequent “go” or “trigger” signal. The description of delay-period activity in PFdl appeared very early in the history of behavioral neurophysiology (Fuster and Alexander 1971; Kubota and Niki 1971; Fuster 1973), and, in accord with the maintenance-memory theory, some PF cells appear to buffer activity representing remembered information, even when distracting stimuli appear during the delay period (di Pellegrino and Wise 1993b; Miller et al. 1996; Moody et al. 1998). Although the interpretation of delay-period activity in terms of the short-term memory of a stimulus has a long history, many studies have explored alternatives.

Neurophysiological experiments designed to explore alternatives to the maintenance-memory interpretation of delay-period activity first attempted to dissociate sensory from motor signals. These studies showed that PFdl neurons preferentially reflected sensory signals, which supported the idea that these neurons encode stimulus memory over the short term. For example, one influential study used the “antisaccade” task (Funahashi et al. 1993b), in which a stimulus in one direction (from a central fixation point) instructed an eye movement in the opposite direction. More than twice as many PFdl neurons represented the location of the sensory stimulus as represented the target (or direction) of movement. In another experiment, when a given spatial cue guided two different reaching movements, motor factors affected PFdl neurons only rarely and weakly compared to neurons in the premotor cortex (di Pellegrino and Wise 1993b), especially when viewed at a population level (Wise et al. 1996a). These results supported the idea that more delay-period activity in PFdl reflected the memory of sensory cues than represented motor preparation or movement targets, but did not explore other alternative interpretations of delay-period activity.

Neuroimaging studies have provided support for some of these alternatives. At first, neuroimaging studies appeared to back the maintenance-memory theory of PF function, which bolstered the interpretation of PF's delay-period activity in the context of that theory. After an initial period of nearly uniform support, however, subsequent neuroimaging studies have suggested that PFdl plays a role in aspects of attention and other functions instead of, or in addition to, maintenance memory. Indeed, one recent report disputed whether PF plays any role in short-term memory at all. To quote the investigators, “no part of frontal cortex, including PF, stores mnemonic representation[s] . . . reliably across distracted delay periods. Rather, working memory storage . . . is mediated by a domain-specific network in posterior cortex” (Postle et al. 2003). Passingham and his colleagues have used the phrases *attention to action, attention to intention,* and *attentional selection* to describe certain PFdl functions (Rowe et al. 2000; Rowe and Passingham 2001). Petrides and his colleagues have, likewise, emphasized a role for PFdl in monitoring items in memory (Owen et al. 1996; Petrides et al. 2002). These alternative views of PF function point to a role in top-down control of attention and are supported by other neuroimaging and neuropsychological findings implicating PF in attentional functions (see Discussion).

In sum, then, neuroimaging and neuropsychological findings bring into question the interpretation of PFdl's delay-period activity mainly in terms of maintenance memory. Previous neurophysiological experiments have ruled out motor factors, such as motor planning and the representation of the targets of movement, for most of PFdl's delay-period activity, but have typically lacked control over spatial attention. The present experiment tested an alternative to the maintenance-memory interpretation of PFdl's delay-period activity by pitting the representation of a remembered location against the representation of an attended location, when either location could serve as the target of an upcoming saccadic eye movement.

## Results

**Figure 1:**
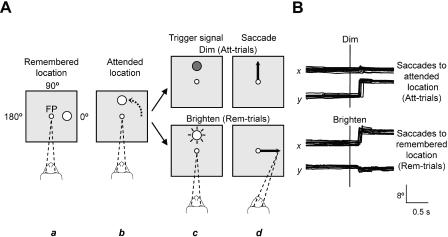
Task and Behavior Behavioral task (A) and representative horizontal and vertical eye position records (B). (A) Each trial began when the monkeys pressed a button to make a fixation point (FP) appear at the center of the video monitor. Some time after the monkeys fixated the FP (dashed lines), a gray circle (depicted here as white) appeared at one of four peripheral locations. The figure illustrates its appearance at the 0° location (part a). The monkeys had to remember this location later in the task; hence we termed it the remembered location. On most trials, the circle subsequently revolved around the FP to a different location, as the monkeys maintained central fixation. The figure illustrates its termination at the 90° location (part b). A small change in the circle's luminance (part c) signaled the monkeys where to look next. This cue persisted for 150 ms, then disappeared. Because the monkeys depended on this subtle and brief cue for both timing and targeting information, we termed this the attended location. If the circle dimmed (dark gray, part c, top), the monkeys had to make a saccade to the attended location (Att-trials, part d, top). If the circle brightened (starburst, part c, bottom), the monkeys had to make a saccade to the remembered location (Rem-trials, part d, bottom). After saccade initiation, the central FP disappeared and, if the monkeys made a saccade to the correct location, a new FP appeared there (not shown). The monkeys had to fixate the new FP and, after it dimmed, release the button to produce a fruit juice reward. (Monkey drawing courtesy of Dr. Michael Shadlen.)

Two monkeys performed the task depicted in Figure 1A. Briefly, the monkeys maintained fixation on a spot presented at the center of a video screen, called the *fixation* point. A solid gray circle then appeared at a fixed distance from the fixation point in any one of the four cardinal directions (Figure 1A, part a): left, right, up, or down from center. Next, as central fixation continued, the gray circle revolved clockwise or counterclockwise around the fixation point, moving along a circular trajectory (arrow in Figure 1A, part b). It then stopped at one of the four cardinal directions from center, after having revolved 90°, 180°, 270°, or 360° (Figure 1A, part b). After a variable delay period of 1.0–2.5 s, the circle brightened or dimmed for 150 ms (Figure 1A, part c) and then disappeared (Figure 1A, part d). The change in the circle's brightness served as the trigger signal for a saccadic eye movement (arrows in Figure 1A, part d). On control trials, the circle either did not move or revolved 360° and stopped at its initial location for that trial. During those trials, both dimming and brightening of the circle instructed a saccade toward its location. During other trials, dimming and brightening of the circle guided both the timing of the response and the choice between two alternative saccade targets.

Brightening of the circle indicated that the monkeys should make a saccade to the circle's initial location on that trial, which the monkeys had to remember in order to perform the task correctly (Figure 1A, parts c and d, bottom). Accordingly, we called these trials *remembered-location trials* (Rem-trials). Dimming of the circle signaled that the monkeys should make an eye movement to its current location (Figure 1A, parts c and d, top). We called these trials *attended-location trials* (Att-trials), for the following reasons. As a key feature of the experimental design, the circle's brightness changed only subtly and remained visible in its new form only briefly. Because the monkeys could not predict whether the circle would brighten or dim and because that subtle, short-lived event provided essential information about the time and target of the response, the monkeys had to attend to the circle intently during the period preceding the trigger signal. As a result of the central fixation requirement, this attention was necessarily covert, although it seems likely that the monkeys would have attended overtly to the circle (i.e., fixated it), had they been allowed to do so. Indeed, the monkeys did so during training. The Discussion takes up the issues of divided attention, multiple motor plans, default motor plans, and other interpretational issues.

By varying the final location of the circle from trial to trial, we could test for significant spatial tuning for attended locations, and by varying the initial location of the circle, we could test for significant spatial tuning for remembered locations. In addition, we tested the monkeys' performance in a “no-memory” condition, which had the same the sequence of events as in the standard version of the task. In the “no-memory” condition, however, the initial location of the circle remained marked by a stationary stimulus identical to the circle that revolved around the fixation point.

### Behavior

Figure 1B shows selected eye-position records, matched to the trials illustrated in Figure 1A. Table 1 shows that both monkeys achieved a high level of performance on this challenging task. For Rem-trials, these data show that the monkeys remembered the circle's initial location, and—because they could not know the trial type in advance of the trigger signal—they must have also done so for Att-trials.

**Table 1:**
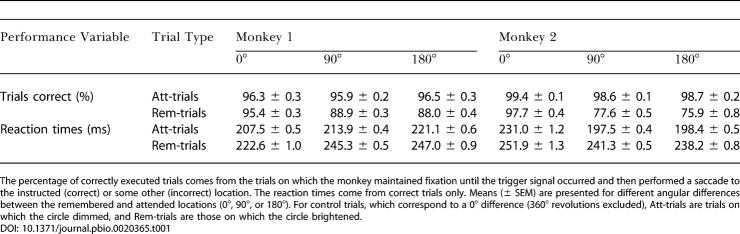
Task Performance and Reaction Times for Each Monkey

The percentage of correctly executed trials comes from the trials on which the monkey maintained fixation until the trigger signal occurred and then performed a saccade to the instructed (correct) or some other (incorrect) location. The reaction times come from correct trials only. Means (± SEM) are presented for different angular differences between the remembered and attended locations (0°, 90°, or 180°). For control trials, which correspond to a 0° difference (360° revolutions excluded), Att-trials are trials on which the circle dimmed, and Rem-trials are those on which the circle brightened

Table 1 also shows the reaction times for each monkey. Taking the two monkeys together, saccades to the remembered location began approximately 36 ms later than those to the attended location, a difference that was highly significant (Wilcoxon rank sum test, *p* < 0.001). We can only speculate about the cause of this difference, but reaction times on Rem-trials may have been longer because attention had to be disengaged from the circle's location and reoriented to the remembered one prior to the response. For the “no-memory” condition (not given in Table 1), reaction times for Att-trials increased approximately 16 ms compared to the standard version of the task, whereas reaction times for Rem-trials decreased approximately 22 ms (both highly significant differences, Wilcoxon rank sum test, *p* < 0.001). These data are consistent with the idea that each of the two marked locations attracted attention in the no-memory condition, whereas the monkeys directed most of their covert attentional resources to the attended location in the standard version of the task. We acknowledge, however, that there are other interpretations of these data. On control trials, for example, when the saccade was always toward the circle, saccade initiation was approximately 18 ms slower when the circle brightened (as it did on Rem-trials) than on trials when it dimmed (as it did on Att-trials). Thus, factors other than the orientation of attention probably contributed to reaction-time differences.

### Single-Neuron Analysis

**Figure 2:**
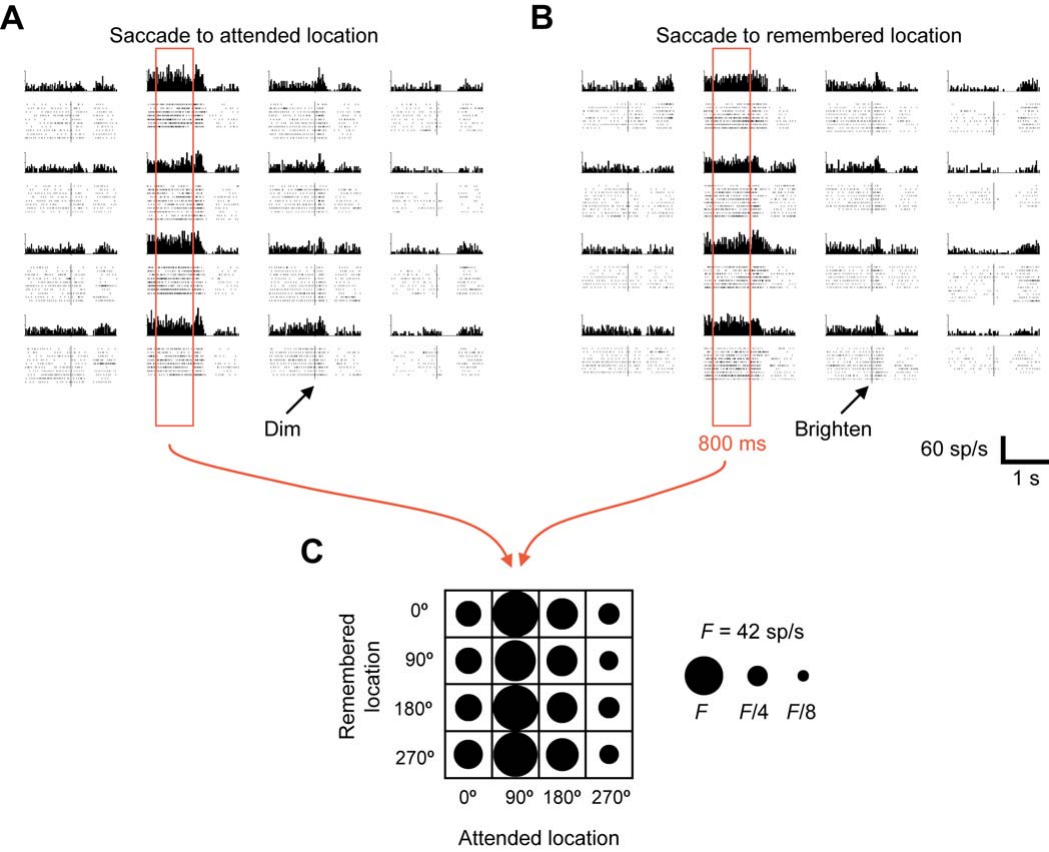
Example Neuron Representing the Attended Location In (A–C), the four rows correspond to different remembered locations and the four columns to different attended locations (see key in [C]). (A and B) PETHs and raster displays aligned on the trigger signal (vertical line). In the rasters, each dot represents a neuronal spike, and each line of dots shows a sequence of spikes during a single behavioral trial. (A) Trials in which the stimulus dimmed and the monkey made a saccade to the attended location (Att-trials). (B) Trials in which the stimulus brightened and the monkey made a saccade to the remembered location (Rem-trials). The activity of this neuron depended on where the monkey attended, with a preferred location of 90°. Note the large variation in firing rate from column to column (across the attended locations) and relative constancy of rate within columns (across remembered locations). (C) Compact representation of spatial tuning pattern shown in (A) and (B), combined. Each circle's area is proportional to the average firing rate during the 800-ms period immediately preceding the trigger signal (red rectangle in [A] and [B]). Note that the major diagonal of this firing-rate matrix, running from the upper left to the lower right corner, corresponds to the control trials, which lacked a memory requirement. F, maximal firing rate; sp/s, spikes per second.

Figure 2 illustrates the activity of a neuron tuned to the attended location during the delay period. Only activity collected during correctly executed trials appears in any of the analyses presented in this report. The figure shows histogram and raster displays of neuronal activity aligned on the trigger signal for Att-trials (Figure 2A) and Rem-trials (Figure 2B), arranged in the form of a matrix, as illustrated and labeled in Figure 2C. Delay-period activity, enclosed by the red rectangles in Figures 2A and 2B, varied with the attended location (columns), but not with the remembered location (rows). The firing rate during the delay period was highest when the monkey attended to the 90° location (up from screen center, see Figure 1A, part b). We called this the cell's *preferred location.* The lowest firing rate occurred when the monkey attended to the 270° location, termed the *least preferred location.*

For each neuron, we assessed the extent of spatial tuning for the attended location with an index called *attended-location index (I_Att_),* which measured the variability in discharge rate among attended locations. We assessed the extent of spatial tuning for the remembered locations with a related index called *remembered-location index (I_Rem_)* (see Materials and Methods). A neuron was considered spatially tuned if *I_Att_, I_Rem_,* or both significantly exceeded 1.0 (randomization test, *p* < 0.01; see Materials and Methods). We classified neurons as *attention cells* if *I_Att_* attained statistical significance but *I_Rem_* did not, as *memory cells* for the opposite result, and as *hybrid cells* if both indexes showed statistical significance.

**Figure 3:**
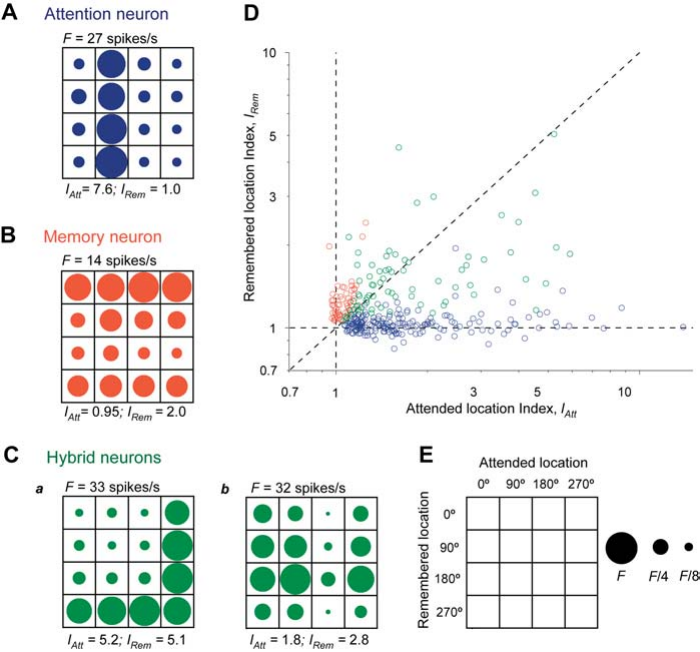
Example Firing Rate Matrices and a Scatter Plot of Tuning Indexes PFdl neurons with different classes of spatial tuning. Firing rate matrices (A–C) in the format of Figure 2C; (E) gives the key. Tuning selectivity indexes *(I_Att_* and *I_Rem_)* and firing rate scale *(F)* appear adjacent to each firing rate matrix. (A) A neuron tuned to the attended location (different from the cell shown in Figure 2). (B) A neuron tuned to the remembered location. Its firing rate primarily varied across rows. (C) Two cells tuned to both the attended and remembered locations (hybrid neurons). One neuron (part a) exhibited a high firing rate when either the attended or remembered location was at 270°. The other neuron (part b) showed its highest activity when the remembered location was at 180°, but was inhibited when that was the attended location. (D) Scatter plot of spatial tuning indexes for attended *(I_Att_)* and remembered *(I_Rem_)* locations for each spatially tuned neuron in both monkeys. The different neuronal classes are color coded as in (A–C): blue corresponds to attention cells, red to memory cells, and green to hybrid cells.

Figure 3A–3C shows examples of an attention cell, a memory cell, and two hybrid cells. (Figures S1–S3 show the trial-by-trial activity for each of these four cells, both before and after the trigger signal.) Neurons tuned to the attended location (attention cells) dominated the neuronal sample in both monkeys, comprising 61% of cells spatially tuned during the pretrigger delay period (Table 2). Neurons tuned to the remembered location (memory cells) made up 16% of the spatially tuned neurons, and those tuned to both locations (hybrid cells) amounted to 23%. For 27% of the hybrid cells, the attended and remembered locations associated with the highest firing rate were the same (Figure 3C, part a); in the remaining 73% of the hybrid cells, these preferred locations differed (Figure 3C, part b).

**Table 2:**
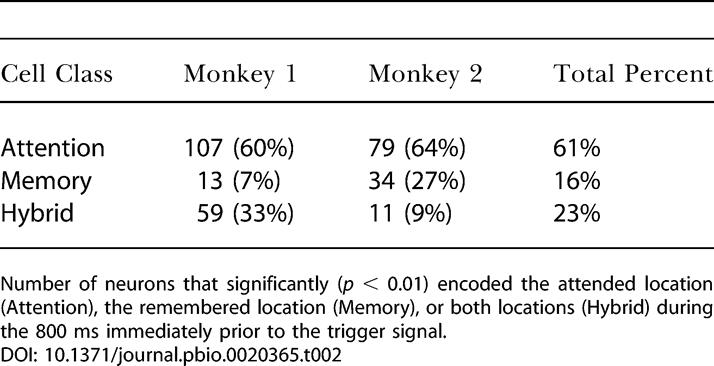
Cell Classification

Number of neurons that significantly (*p* < 0.01) encoded the attended location (Attention), the remembered location (Memory), or both locations (Hybrid) during the 800 ms immediately prior to the trigger signal

Figure 3D illustrates the degree of tuning for both the attended *(I_Att_)* and remembered (*I_Rem_*) locations. Each data point on the scatter plot represents a single spatially tuned neuron (both monkeys combined). Tuning for the remembered location (red symbols) was both weaker and less frequent than tuning for the attended location (blue symbols). Note that hybrid cells (green symbols) fill most of the space between the other two classes and that relatively few cells represent a single location exclusively. For example, many of the neurons classed as memory cells show some sensitivity to the attended location, albeit not a statistically significant one by the test that we employed. For the entire group of spatially tuned neurons (*n* = 303, both monkeys and all three cell classes combined), the mean selectivity indexes (± SEM) for the attended and remembered locations were *I_Att_* = 1.84 ± 0.08 (median = 1.39, interquartile range [IQR] = 0.73) and *I_Rem_* = 1.21 ± 0.02 (median = 1.08, IQR = 0.23), which differed significantly at the *p* < 0.001 level (Wilcoxon matched-pairs test). Table 3 shows comparable data for each cell class and Figure S4 gives similar data for various combinations of these classes. The selectivity for the attended location also exceeded that for the remembered one when expressed in terms of firing rates. For the attended location, the difference in firing rate between the preferred and least preferred locations averaged 8.8 ± 0.5 spikes/s, which was significantly greater than the 5.3 ± 0.3 spikes/s for the remembered location (Wilcoxon matched-pairs test, *p* < 0.001).

**Table 3:**
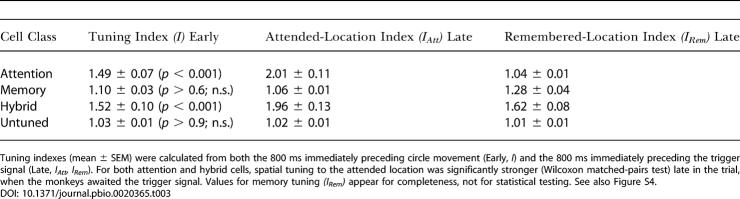
Spatial Tuning Indexes Early Versus Late in the Trial

Tuning indexes (mean ± SEM) were calculated from both the 800 ms immediately preceding circle movement (Early, *I*) and the 800 ms immediately preceding the trigger signal (Late, *I_Att_, I_Rem_*). For both attention and hybrid cells, spatial tuning to the attended location was significantly stronger (Wilcoxon matched-pairs test) late in the trial, when the monkeys awaited the trigger signal. Values for memory tuning *(I_Rem_)* appear for completeness, not for statistical testing. See also Figure S4

We examined whether these results merely reflected the presence of a stimulus in the monkey's visual field and found strong evidence to the contrary. We compared tuning for the circle's location during the 800 ms before the circle started moving (called the *early* period) and during the last 800 ms of the delay period, immediately prior to the trigger signal (the *late* period). (Figures S5 and S6 show activity during a slightly different early period than measured here, but they illustrate the same basic result.) Despite the fact that the sensory inputs were identical in screen-centered, allocentric, retinocentric, fixation-centered, head-centered, and body-centered coordinates, the activity of PFdl neurons and their degree of spatial tuning differed in these two task periods. This result rules out a purely sensory response. For the entire PFdl sample, the late tuning index (1.29 ± 0.03) significantly exceeded the early one (1.16 ± 0.02; *p* < 0.001; Wilcoxon matched-pairs test). This measure is devoid of any bias caused by a cell's tuning properties in one task period or the other, but it includes the contribution of the spatially untuned cells. When we restricted the comparison to neurons that had any type of significant spatial tuning, in either the early or late periods, the late tuning index (1.76 ± 0.07) continued to exceed the early one (1.42 ± 0.05) significantly (*p* < 0.001). Most important, we obtained similar results for neurons with significant tuning to the circle's location, which characterizes attention and hybrid cells (1.83 ± 0.08 late versus 1.46 ± 0.05 early; *p* < 0.001). Table 3 and Figure S4 present this analysis for all cell classes, alone, and in various combinations. Note that these indexes do not reflect a generalized increase in firing rate: They were normalized to remove the effects of firing rate per se. The section entitled Population Analysis presents a confirmatory result in terms of activity levels. Further confirming this result on a cell-by-cell basis, significant spatial tuning to the circle's location occurred more frequently during the late delay period (256 attention and hybrid cells) than during the early one (194 cells, of which 41 lost their spatial tuning in the late period). Thus, the representation of the circle's location in PFdl grew stronger around the time of the trigger signal, when it was important for the monkeys to attend to the circle. These findings rule out the mere presence of the circle in something akin to a visual receptive field as a complete account of the tuning of attention and hybrid cells.

### Histological Analysis

**Figure 4:**
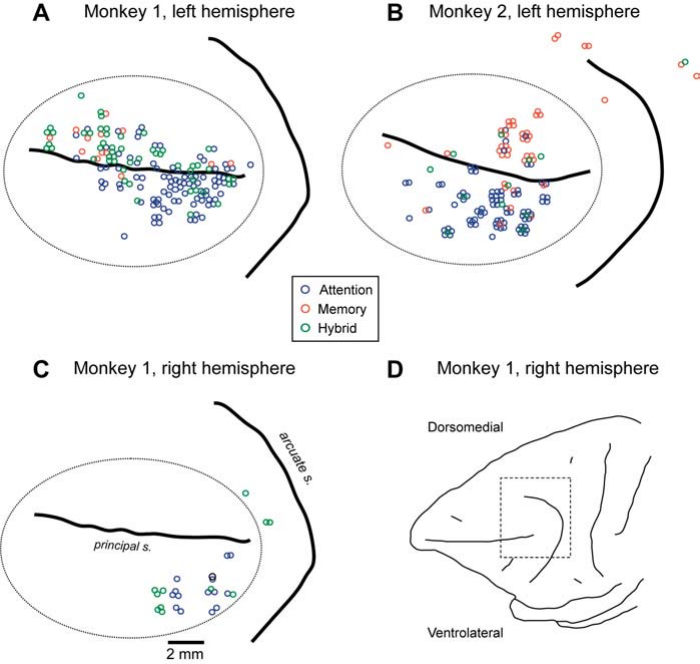
Surface Projections Showing the Location of Neurons in Each Class All hemispheres are displayed so that rostral is to the left and dorsomedial is up. Reconstructed surface projections of the left hemispheres of monkey 1 (A) and monkey 2 (B). (C) Surface projection of the (inverted) right hemisphere of monkey 1. (D) A lateral view of the hemisphere shown in (C), with the region included in (C) approximated by the dashed box. The dotted ellipse encloses the cells deemed to lie inside the PFdl by histological analysis, but does not correspond to the cytoarchitectonic boundaries of area 46.

**Figure 5:**
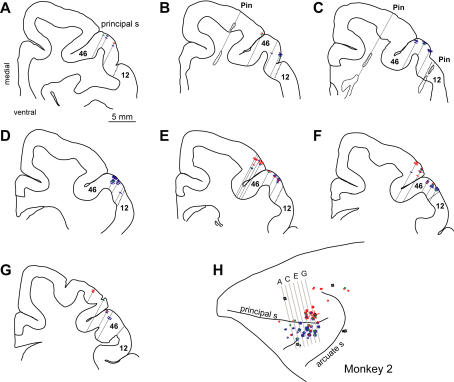
Section Reconstructions for Monkey 2 (A–G) Coronal sections taken at the planes indicated in the surface drawing (H). Dashed lines mark the borders between PFdl (area 46) and area 12. Solid lines show the tracks of the marking pins (irregular outlines in sections [B] and [C]) and the estimated location of electrode penetrations. Colored hash marks show the estimated depth of neurons in each class, using the same color code as in Figures 3 and 4. Longer hash marks indicate simultaneous recordings of more than one neuron of the same class. (H) Lateral view of left PF depicting surface projections of spatially tuned neurons. Black circles show the locations of pin holes used for localization, and gray squares show their predicted locations.

Figures 4 and 5 show the locations of the cells in each class: Figure 4 as a function of electrode-penetration sites for both monkeys and Figure 5 as section reconstructions for monkey 2. The attention cells were concentrated more ventrolaterally than either the memory or the hybrid cells. Neurons located ventrolateral to the fundus of the principal sulcus (*n* = 551) were predominantly attention cells (28% to 2% memory and 5% hybrid cells, with 65% lacking spatial tuning, both monkeys combined). Neurons dorsomedial to the fundus (*n* = 412) fell into the three cell classes approximately equally (8% attention, 9% memory, and 10% hybrid cells, with 73% lacking spatial tuning). These regional differences within PFdl were highly significant for each monkey (p < 0.0001, χ^2^ test). Cells with significant memory signals (memory and hybrid cells, combined) composed 70% of the spatially tuned population in dorsomedial PFdl, but only 20% in ventrolateral PFdl.

Based on a cytoarchitectonic analysis conducted on two of the three hemispheres, all of the cells situated ventrolateral to the fundus of the principal sulcus were located within area 46 and none were located in area 12. The area 46/12 architectonic boundary was first described by Walker (1940) and was subsequently confirmed with different methods (Preuss and Goldman-Rakic 1991). This boundary could be discerned in both monkeys as a distinct thinning of the internal granular layer in area 12 compared to area 46 and a more substantial departure in that area from the classic, homotypical appearance typical of area 46. The reconstructed location of recording sites showed that the small group of cells located caudomedially in both monkeys (see Figure 4B and 4C) was located in the postarcuate cortex (area 6) and in area 8, as indicated by the agranular and dysgranular cytoarchitecture of these two regions, respectively. This small group of cells was eliminated from the present analysis.

### Population Analysis

**Figure 6:**
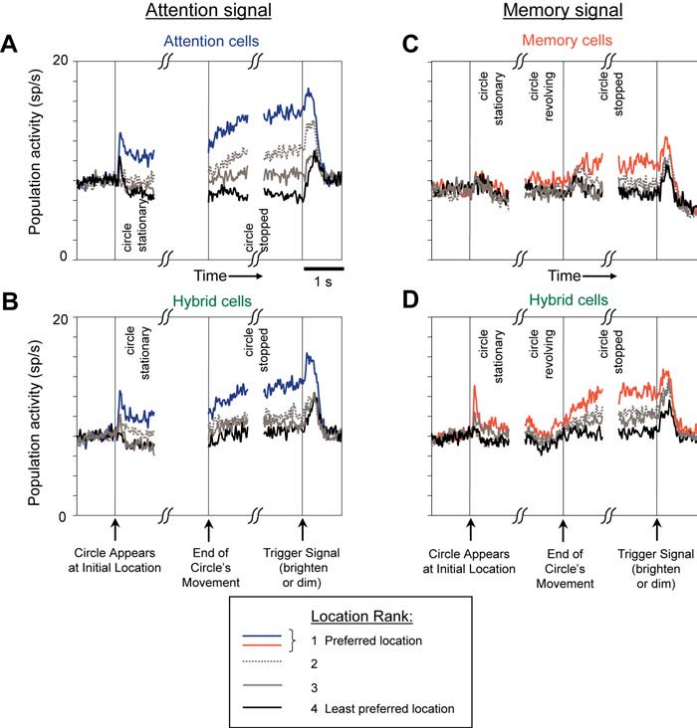
Attention and Memory Signals in Population Histograms (A) and (B) Representation of an attention signal by attention cells (A) and hybrid cells (B). (C) and (D) Representation of a memory signal by memory cells (C) and hybrid cells (D). In each panel, activity is shown centered on the appearance of the circle (left vertical line), on the time that the circle stopped moving (middle vertical line), and on the trigger signal (right vertical line). Attention and memory signals are reflected in the degree of separation in the average population histograms for different ranks. In (A) and (B), the data for the period immediately prior to the end of the circle's movement have been eliminated because the circle came from different initial locations.

Figure 6 displays the degree of spatial tuning for the different cell classes in the form of population histograms. The analysis of attention tuning (Figure 6A and 6B) used the 800 ms immediately preceding the trigger signal to measure mean firing rates for different attended locations. We excluded control trials from this analysis. These rates were then ranked from the largest (i.e., the preferred attended location) to the smallest (the least preferred location). For each neuron, the preferred location chosen by this analysis was designated as preferred for all task periods displayed in the population histograms. (Similar results were obtained when the ranking was done for each individual task period.) The left side of the figure shows the mean attention signal for both attention (Figure 6A) and hybrid (Figure 6B) cells. After a transient response to the appearance of the circle (at a latency of approximately 100 ms), neuronal activity in both of these cell classes remained elevated when the circle stopped at the preferred location (blue curve) and became slightly suppressed when it was at the least preferred location (black curve).

The right side of Figure 6 shows the mean memory signal for memory (Figure 6C) and hybrid (Figure 6D) cells. These population histograms were calculated on the basis of preferred remembered locations, ranked according to the pretrigger modulation. This location was then designated “preferred” for all task periods displayed in the plots. For memory cells (Figure 6C), the population averages were almost identical when the circle remained stationary at its initial location and that location did not yet need to be remembered. That is, on average it did not matter noticeably whether the circle initially appeared at a cell's preferred location or at its least preferred location (Figure 6C, red versus black curves). This finding is somewhat surprising because prior studies suggested that PFdl's memory cells had activity that began shortly after stimulus onset and continued throughout the delay period. In our memory cells, spatial tuning did not develop to any appreciable extent until after the circle began revolving around the central fixation point. This result shows that tuning to the remembered location developed during the trial and was not a simple replica of the tuning pattern during the initial presentation of the circle. Hybrid cells (Figure 6D) exhibited a weak spatial signal following the appearance of the circle consistent with their memory tuning prior to the trigger. Note that after the circle stopped moving, memory cells showed less of a difference between preferred and least preferred locations than did attention cells (Figure 6C versus 6A). This finding supports the results presented in Tables 2 and 3 and Figure 3D, which show a predominance of nonmemory signals (see also Figure S4).

Population representations of the attended and remembered locations were further analyzed using a neuron-dropping analysis. Neuron-dropping curves express the strength of spatial tuning as the ability to estimate a spatial variable from the activity of a neuronal ensemble, as a function of ensemble size. We randomly selected an ensemble from the population of recorded PFdl neurons and used a single trial of activity from each cell to estimate both the attended and remembered locations. The findings of the neuron-dropping analysis agree with those from the analysis of single-cell activity and the population histograms and thus provide independent support. However, neuron-dropping analysis offers several advantages over the population histograms, in addition to providing confirmation of those results. In neuron-dropping, the estimation of either an attended or remembered location does not depend on any assumptions about the nature of the spatial tuning curve or the relative importance of very active cells versus those showing less activity. It does not ascribe any special significance to increases in activity relative to baseline (excitation) versus decreases (inhibition) or to the most preferred and least preferred locations. Each cell's activity contributes to the population estimation for all locations regardless of the direction of its modulation relative to baseline and whether that modulation significantly differs from baseline levels. Furthermore, the computation makes no assumption about any relationship between tuning for attended locations and remembered ones. This analysis also has the advantage that its results are expressed as a percentage of correct estimations by the neuronal ensemble, thereby facilitating comparison with the monkeys' performance, which in this experiment always exceeded 75% correct and sometimes approached 100% (Table 1).

**Figure 7:**
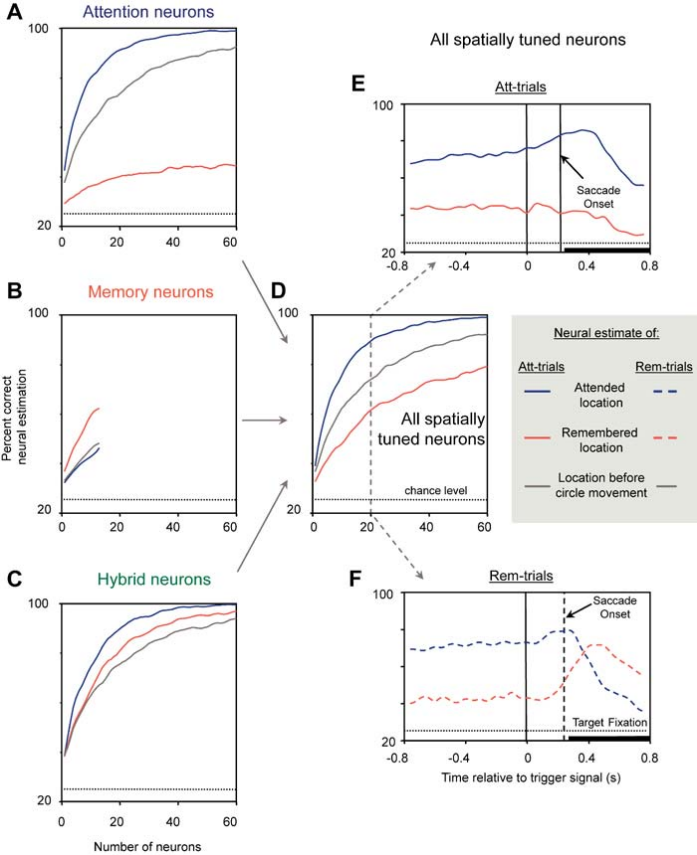
Neuron-Dropping Curves for Different Subpopulations of PFdl Neurons in Monkey 1 Each curve represents the percentage of correct single-trial estimations of location as a function of the number of neurons in the assembled populations. The curves show predictions of the attended locations (blue lines) or remembered locations (red lines) during the 800 ms immediately preceding the trigger signal, after the circle had stopped revolving around the central fixation point. Also shown is the estimation for the 800-ms period immediately preceding the onset of the circle's movement (gray lines). The dotted line indicates the chance level of estimation, 25% correct. Neuron-dropping curves are shown for neurons tuned to the attended location (A), the remembered location (B), both locations (C), and all spatially tuned neurons (D). (E) and (F) Dynamic changes in estimations of the attended (blue) and remembered (red) locations for 20 spatially tuned neurons (marked by the dashed gray line and arrows), using a 200-ms sliding window. Dashed and solid lines in (E) and (F) are shown for consistency with Figure 8. Note that the estimations in (D) are higher than in (E) and (F) because the former is based on an 800-ms interval, and the latter are based on only a 200-ms interval.

Figure 7 shows the neuron-dropping curves for each cell class (A–C) and all spatially tuned neurons combined (D) in monkey 1. Neuron-dropping curves for monkey 2 showed similar results, and Figure S7 presents the data for both monkeys combined. As expected, the neuron-dropping curves computed for attention cells yielded much better estimations of the attended location than the remembered one (see Figure 7A, blue versus red curves). Note, however, that the attention cells also provided a better-than-chance estimation of the remembered location. This result reflects the fact that many cells with significant tuning for the attended location also showed some tuning for the remembered location (see blue data points in Figure 3D with *I_Rem_* > 1.0). Figure 7A also confirms the comparison of activity early versus late in the trial (blue versus gray curves), providing further evidence against a purely sensory account of this subpopulation's activity. Also as expected, memory cells yielded a better estimation of the remembered location than the attended one (Figure 7B, red versus blue curves), but these cells, too, yielded a fairly reliable estimation of the other spatial variable. Neuron-dropping curves for hybrid neurons showed comparable estimations for both locations (Figure 7C). When all spatially tuned neurons were combined (Figure 7D; see also Figure S7D), the resultant neuron-dropping curves showed that PFdl activity was a much more reliable estimator of the attended location than the remembered one.

The same analysis was applied to the ventromedial and dorsolateral regions within the PFdl, described in the section entitled Histological Analysis, above (not shown). The ventrolateral subpopulation of PFdl neurons (see Figure 4A–4C) overwhelmingly represented the attended location. The dorsomedial subpopulation represented both locations comparably, with estimation of the attended location being slightly better in one monkey and estimation of the remembered location being slightly better in the other. Of the two subpopulations, the dorsomedial neurons showed a more reliable estimation of the remembered location.

**Figure 8:**
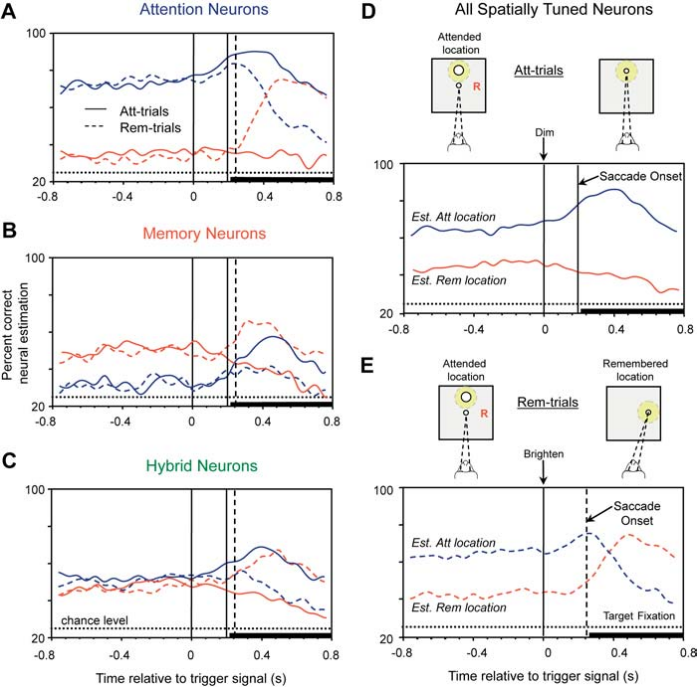
Time-Dependent Changes in Estimating the Attended Location and Remembered Location, for Both Monkeys Combined Solid lines, trials in which the monkeys made a saccade to the attended location; dashed lines, trials in which the monkey made a saccade to the remembered location. Blue lines, estimation of the attended location; red lines, estimation of the remembered location. All records are centered on the onset of the trigger signal (using data for the 200 ms prior to that time). Vertical lines at t > 0 show the average saccade latency on Att-trials (solid) and Rem-trials (dashed). The thick bar at the bottom of the plots shows the approximate onset of the peripheral fixation spot, which the monkeys continued fixating beyond the limit of the plot. Estimations for each monkey were calculated using the same methods as for Figures 7E and 7F, except that the ensemble size for monkey 2 was 60 neurons. This number of neurons was chosen to avoid ceiling effects (i.e., 100% correct). The plotted curves show the average for the two monkeys. Location estimations for attention (A), memory (B), hybrid cells (C), and all spatially tuned neurons on Att-trials (D) and Rem-trials (E). Above the plots are schematic depictions of an example trial, of the type illustrated in Figure 1A. The red “R” marks the remembered location. In both D and E, prior to the trigger signal (left schematic), the monkeys fixated (dashed lines) centrally and covertly attended to the circle (yellow spot at the attended location). During this period, estimation of the attended location exceeded that of the remembered location. Following the saccade, the monkeys fixated a peripheral light spot (right schematic) and attended to this target to detect when it dimmed. On Att-trials (D), the monkeys' gaze shifted to the attended location, and the ensemble's estimation of this now overtly attended location improved (solid blue curve), while the representation of the now irrelevant, remembered location gradually decayed (solid red curve). On Rem-trials (E), the monkeys' gaze (dashed lines) and focus of attention (yellow spot) shifted to the (previously) remembered location. The estimation of this location consequently improved (red dashed curve), while the estimation of the previously attended (and now irrelevant) location gradually decayed. Abbreviations: Att, attended; Rem, remembered.

We also used a neuron-dropping analysis to examine the ensemble's properties during response selection and execution. Figure 7E and 7F show these time-dependent neural-estimation curves for monkey 1; Figure 8 does so for both monkeys combined. Note from Figure 7D–7F that the time-estimation curves come from a random sample of neurons, much smaller than the sampled population, to avoid the effects of signal saturation. The estimations at each time point reflect activity averaged over the previous 200 ms. Prior to the trigger signal, the estimation of the attended location (blue curves in Figures 7E, 7F, 8D and 8E) was superior to that of the remembered location (red curves) for all spatially tuned neurons, as well as for attention cells (Figure 8A). This finding is consistent with the greater number and stronger spatial tuning of attention than memory cells.

On Att-trials, the estimation of the attended location (solid blue curves in Figures 7E and 8A–D) improved following the dimming of the circle and remained elevated during the saccade to that location. This improvement continued for the initial 200 ms of fixation there. Then the signal decreased. Note that the monkey maintained fixation at the target location for at least 1.0 s after the saccade. In contrast, the estimation of the remembered location on Att-trials (solid red curves) gradually decreased following the trigger signal. The fading of this representation most likely reflected the fact that the remembered location was no longer behaviorally relevant.

On Rem-trials (dashed curves in Figures 7F, 8A–8C, and 8E), the circle's brightening instructed a saccade to the remembered location (marked by the red “R” in Figure 8E). We expected that redirecting attention toward the saccade target (yellow spot in Figure 8E, right) would degrade the neuronal representation of the formerly attended location and improve the representation of the formerly remembered—but eventually fixated—one. The estimation of the attended location initially improved on Rem-trials following the trigger signal there (blue dashed curves in Figures 7F, 8A–8C, and 8E). However, in accord with our expectation, that estimate decreased dramatically in accuracy after saccade onset, as the attended location became behaviorally irrelevant. In contrast, the estimation of the formerly remembered (and soon to be fixated) location (red dashed curves) improved sharply (Figure 8E), especially in attention cells (Figure 8A). Thus, PFdl neurons became more reliable encoders of that location. Given that these averages “look back” 200 ms, this development must have preceded the saccade.

On both Att-trials and Rem-trials, the neuronal ensemble remained a reliable indicator of the saccade target relatively long after the target had been acquired (see solid blue and dashed red curves in Figures 7E, 7F, and 8). This signal might encode the fixated location, which could be important for monitoring performance, as suggested for nearby areas of frontal cortex (Stuphorn et al. 2000; Ito et al. 2003). Alternatively, the saccade target may have been represented because the monkeys attended to the fixation spot at this location, so that when it dimmed they could quickly release the button to produce their reward (see Materials and Methods, below, for a description of that aspect of the task).

## Discussion

In tasks involving short-term memory requirements, delay-period activity in PFdl has consistently been interpreted in terms of the maintenance-memory theory of PF function (e.g., Funahashi et al. 1989; Romo et al. 1999; Constantinidis et al. 2001), despite the existence of viable alternatives. However, our results show that much of PFdl's delay-period activity in such tasks reflects nonmemory functions. Accordingly, the maintenance-memory theory of PF function (Goldman-Rakic 1987, 1990), taken to its extreme, fails to account for PFdl's delay-period activity. Indeed, we found that, compared to the remembered location, the attended location was more frequently and more robustly encoded at both the neuronal and population levels. The present results thus support extensive neuropsychological (Rueckert and Grafman 1996; Stuss et al. 1999; Koski and Petrides 2001, 2002) and neuroimaging (Corbetta et al. 1993; Gitelman et al. 1999; Kastner et al. 1999; Rosen et al. 1999; Cabeza and Nyberg 2000; Hopfinger et al. 2000, 2001; Vandenberghe et al. 2000; Astafiev et al. 2003; Small et al. 2003; Thiel et al. 2004; Woldorff et al. 2004) research that points to a much more general role for PF than encompassed by the maintenance-memory theory, including the top-down control of selective attention.

### Interpretational Issues and Limitations

The present experiment is the first neurophysiological study to achieve a degree of independent control over both spatial attention and spatial memory, so a detailed consideration of both its advantages and limitations is in order. A complete dissociation of these two spatial variables is probably impossible, but we achieved this goal to a considerable degree. Our experimental design, however, has several limitations and raises a number of questions. For example, is what we call attention really attention? We have elaborated on our usage of the term *attention* in the Results section. Although we did not quantify the degree of attention, it seems to us a reasonable assumption that the monkeys attended to the circle, given that its brightening or dimming was subtle, brief, and crucial to their correct performance. Moreover, the reaction-time data are consistent with the idea that the monkeys attended to the circle in the period immediately prior to the trigger signal. The remaining interpretational questions to be addressed, then, are: Do monkeys devote any attentional resources to what we call the remembered location? Do they “remember,” in some sense, what we call the attended location? Does the activity we interpret in terms of attention or memory reflect motor factors? And, given that the monkeys could anticipate and predict rewards, do the signals reflect these processes? We address each of these four questions, in turn, in the remainder of this section.

First, although we contend that the monkeys must have devoted substantial attentional resources to the location of trigger signal, this does not necessarily rule out additional covert allocations of attention to the remembered location. However, there was no stimulus or expected signal at the remembered location to warrant the allocation of attentional resources there. In addition, the demands of fixating the central location (overt attention), while attending covertly to a stimulus located in peripheral visual space, make it unlikely that attention was further divided (Hunt and Kingstone 2003; Muller et al. 2003). Accordingly, although we cannot completely rule out the possibility that the monkeys attended to the remembered location during the delay period, it seems implausible that they did so. If one adopts the view that they did, then some or all of the neurons we class as memory cells might instead have activity better interpreted as reflecting some aspect of highly divided attention.

Second, the monkeys were required to remember the place where the circle first appeared on each trial, and their performance shows that they did so. Did they also “remember” the attended location? There is ample precedent for skepticism about the proposition that monkeys are *not* remembering some location. However, there is no basis for assuming a “memory” of a currently visible stimulus. It seems especially unlikely that the monkeys “remembered” the attended location in the context of the requirement that they centrally fixate while attending somewhere and remembering somewhere else.

Third, we cannot rule out the participation of neurons we class as attention or memory cells in a variety of processes involved in preparing or planning the movement or selecting the response target. Prior to the trigger signal, the monkeys may have prepared to make a movement to the remembered location, to the attended location, to both, or to neither. Cisek and Kalaska (2002) have shown that some neurons in the premotor cortex encode a possible movement target before a particular one has been specified, but their experiment has yet to be done for PFdl neurons. In view of prior evidence arguing against interpreting much of PFdl's delay-period activity in terms of motor signals (Funahashi et al. 1989, 1993b; di Pellegrino and Wise 1993b; Asaad et al. 1998; Romo et al. 1999; Constantinidis et al. 2001) and the absence of a contemporary “motor theory” of PF function, the present experiment was not designed to address this issue. Future work along these lines, perhaps combining the design of di Pellegrino and Wise (1993b) with the present one, might be indicated by the present results. We believe, however, that a simple “motor” explanation for most of PFdl's delay-period activity is an unlikely outcome of such studies. A “motor” interpretation probably does, however, account for a small proportion of PFdl's delay-period activity, consistent with the results of Funahashi et al. (1993b). On certain assumptions about a default motor plan, such neurons could have the tuning properties of the hybrid cell illustrated in Figure 3C, part a. It is important to emphasize, however, that the present experiment tested whether the maintenance-memory theory could account for all delay-period activity in PFdl. It cannot. We view this result as supporting an important role for PF in the top-down control of attention. If one takes a motor theory of PF function more seriously than most expert opinion currently does, then it is possible to interpret the present result as indicating a role in context-dependent response or goal selection or in terms of the preparation of movements to remembered targets versus current stimuli. Neither interpretation is consistent with an interpretation of PFdl's delay-period activity entirely in terms of a maintenance-memory function.

Fourth, we need to consider the possibility that the neural signals we observed reflect the prediction or anticipation of reward. Maunsell (2004) has recently pointed out that neural signals interpreted as arising from attention could instead reflect reward anticipation or prediction (and vice versa). In the present study, however, reward-related information processing could not have accounted for the properties of attention cells because, until the trigger signal, one alternative place (the remembered location) was associated with reward to the same degree as the attended location.

### Enhancement Effects

The general term *attention* has been used to cover many disparate concepts, including the effects of attention on sensory processing and the mechanisms that mediate those influences. We emphasize that the present finding differs from previous ones describing effects of attention on phasic, sensory-like responses. Often called the *enhancement effect,* the finding that sensory responses are larger when a stimulus or location is more attended was first described for the superior colliculus (Wurtz and Goldberg 1972) and has been repeatedly demonstrated for many cortical areas, including PFdl (Mikami et al. 1982; Boch and Goldberg 1989; di Pellegrino and Wise 1993a; Rainer et al. 1998; DeSouza and Everling 2004). In some instances, and especially in frontal cortex, the enhancement effect depends on the attended location being the target of a movement (Goldberg and Bushnell 1981), but in other cases it does not (Bushnell et al. 1981). It has often been suggested that the source of attention effects, including the enhancement effect, match enhancement, and related phenomena, depends on signals emanating from PF (Miller et al. 1996; Kastner et al. 1999; Reynolds et al. 1999) or from the frontal eye field (Thompson et al. 1997; Moore and Fallah 2004). The present results are consistent with this idea. They cannot, however, be considered as yet another example of the enhancement effect, which involves attention-dependent augmentation of a phasic sensory response.

### Neurons Encoding Both Attended and Remembered Locations

Most neurons did not encode an attended or remembered location exclusively; rather, they exhibited varying degrees of tuning for both variables. The neuron-dropping curves (see Figure 7) show that attention cells were able to make limited, but above-chance, estimations of the remembered location and vice versa. As can be seen from the spatial tuning indexes in Figure 3D, few individual neurons were pure attention or memory encoders (data points along the axes). Thus, the population of spatially tuned cells can be viewed as a continuum with attention and memory cells at the extremes, and hybrid cells in between.

Interestingly, the neuron-dropping curves for the hybrid cells (see Figures 7C and 8C) showed effective estimation of both the attended and remembered locations. Hybrid neurons with dissimilar preferences for the two locations facilitated such estimations. For instance, the neuron shown in Figure 3C, part b had a low firing rate when the monkey attended to the 180° location and a high firing rate when it remembered that place. Hybrid cells with dissimilar preferences can resolve the ambiguity inherent in cell activity like that illustrated in Figure 3C, part a, which cannot distinguish between attended and remembered locations.

### Previous Neurophysiological Studies

Previous neurophysiological studies of PFdl's delay-period activity have been interpreted in terms of the maintenance-memory theory. However, the lack of control over spatial attention in these studies raises questions about these interpretations. Constantinidis et al. (2001), for example, trained monkeys to make delayed saccades toward the location of the brighter of two visual stimuli that briefly flashed on the video screen. They reported that the activity of PFdl neurons reflected the brightness of the stimuli. Although these authors interpreted their findings as demonstrating a purely sensory-mnemonic function for PFdl neurons, brighter stimuli, being more salient, are well known to attract attention to their location.

Similar problems affect the interpretation of data from the “antisaccade” task (Funahashi et al. 1993b). In their antisaccade task, Funahashi et al. trained a monkey to respond to a stimulus to the left of a fixation point by making a saccade to the right and vice versa. They interpreted their data as demonstrating a function for PFdl in spatial memory because the largest number of neurons reflected the stimulus location rather than the movement target. They showed that during the delay period, when nothing was present on the screen, some neurons reflected where the stimulus had occurred, and these were interpreted as memory cells. Note, however, that where ever the stimulus appeared, whether in antisaccade or prosaccade trials, it served as an attention attractor. If the response to that signal persisted, then interpreting it exclusively as a sensory memory trace would be problematic. Many studies suggest that, for neurons in PF, the history of what has happened or the context in which it happens often affects neuronal activity in an important and persistent way (Rainer et al. 1998; Asaad et al. 2000; Wallis and Miller 2003), sometimes regardless of relevancy (Chen et al. 2001). Such persistent signals can be viewed as components of working memory in a general sense, but not in the narrow sense implied by the concept of maintenance memory.

### Neuroimaging and Neuropsychological Results from Humans

Based on the idea that the principal or exclusive function of PFdl is to support maintenance memory (Goldman-Rakic 1987), many neuroimaging papers on PF, including PFdl, have been interpreted as supporting this theory of PF function (see, for example, Courtney et al. 1996, 1997, 1998; Druzgal and D'Esposito 2003; Inoue et al. 2004). This idea has been defended (Goldman-Rakic 2000), but a number of alternatives have been suggested. For example, several neuroimaging findings support a role for PF in the control of attention, and brain lesion studies also show attentional deficits after damage to various parts of PF (Corbetta et al. 1993; Rueckert and Grafman 1996; Gitelman et al. 1999; Kastner et al. 1999; Rosen et al. 1999; Stuss et al. 1999; Cabeza and Nyberg 2000; Hopfinger et al. 2000, 2001; Vandenberghe et al. 2000; Koski and Petrides 2001, 2002; Astafiev et al. 2003; Small et al. 2003; Thiel et al. 2004; Woldorff et al. 2004; see also a recent review by Wood et al. 2003).

In general, top-down attention has been assumed to result from signals emanating from the frontal cortex and biasing more posterior areas to favor some channels of information over others, and some neuroimaging papers have supported this idea (Chawla et al. 1999; Kastner et al. 1999; Corbetta and Shulman 2002; Nakahara et al. 2002; Pessoa et al. 2003). In addition, a role in attentional selection and the related concepts of attention to action and attention to intention have been stressed as an alternative to the maintenance-memory theory of PF function (Rowe et al. 2000; Rowe and Passingham 2001; Lau et al. 2004). Similarly, monitoring the items in short-term memory has been put forward as a principal function of PFdl, and this also is primarily an attentional function (Owen et al. 1996; Petrides et al. 2002). Along these lines, a recent study by Nobre et al. (2004) indicated that PF plays a role in directing attention to locations within mental representations.

### Neuropsychological Results from Monkeys

Previous research on monkeys has also suggested a role for PF (or nearby parts of the frontal lobe) in the orientation of spatial attention. Welch and Stuteville (1958) produced trimodal (auditory, visual, and tactile) neglect-like effects following ablations in the depths of the arcuate sulcus, including what was likely part of PF (although not PFdl). Rizzolatti et al. (1983) reported neglect for space beyond a monkey's reach after lesions targeting area 8. However, for at least one of the two monkeys they studied, the lesion may have included the area studied here. Deuel and Farrar (1993) also produced neglect-like symptoms by making cortical lesions that included much of the same region, and roughly similar observations have been interpreted as motor neglect (Heilman et al. 1995). PF lesions also caused attention-like deficits in a conditional motor learning task (M.F.S. Rushworth et al., personal communication).

In the context of the present results, the finding that inactivation of parts of PFdl (Funahashi et al. 1993a) produced what were termed “mnemonic scotomas” deserves reconsideration. In that experiment, a transient cue served as the target of a saccade after a delay period. Following local inactivations within PFdl, the monkeys in that study continued to make most of their responses to sites near the cue's remembered location, even with 3-s and 6-s delays after the disappearance of the cue (see their Figures 5, 9, and 13). The monkeys made the vast majority of their responses in the correct direction, but a few saccades fell outside the target zone. This inaccuracy contributed to significantly increased variance in the endpoints of the saccades, and Funahashi et al. (1993a) concluded on this basis that the monkeys were unable to remember the cue's location. We suggest, as an alternative explanation of their results, that their monkeys had a deficit in detecting the stimulus at the cued location, directing attention there, or maintaining their attention at the cued location. Thus, the results interpreted as “mnemonic scotomas” might be better understood as a localized neglect-like phenomenon or some combination of attention and memory deficits. This suggestion finds support in the results of a recent study in humans with PF lesions. Hornak et al. (2004) reported a failure of such patients to pay attention to information on a screen, and this problem accounted for their behavioral deficits. Therefore, the results of Funahashi et al. (1993a) provide little support for either the maintenance-memory theory of PF function or the interpretation of its delay-period activity in terms of that theory.

The present results agree better with those of Rushworth et al. (1997), who found that monkeys could remember nonspatial stimuli across relatively long delay periods after bilateral removal of the part of PF theorized to maintain such memories. The present results also agree with Petrides (2000), who found that PFdl lesions do not affect the short-term memory for objects (as measured by a susceptibility to increasing delay periods), but do cause impairments in the ability to monitor which items have been selected from a group (as measured by a susceptibility to increasing group size).

### Conclusions

The present study reexamined the interpretation of PFdl's delay-period activity in terms of the maintenance-memory theory. We found that other factors are more important than mnemonic ones. The present results do not argue against a short-term memory function for PF, as one among many contributions to behavior. Nor should they lead to the dismissal of interpretations of some delay-period activity in PF, or some neuroimaging signals from that region, in terms of short-term memory. However, spatial memory signals occur less frequently in PFdl than the maintenance-memory theory predicts. Our data thus accord better with neuroimaging and neuropsychological studies indicating that PF plays a major role in attentional selection, including the monitoring of information and actions (Owen et al. 1996; Rowe et al. 2000; Rowe and Passingham 2001; Petrides et al. 2002; Manly et al. 2003; Lau et al. 2004).

How do our findings mesh with the fact that damage to PF appears to produce deficits in short-term memory, as Jacobsen (1935, 1936) first showed nearly 70 years ago? One possibility is that lesion studies speak more to the inability of other areas to compensate for the loss of PF than to the priority of functions within that region. Another is that an attentional deficit would likely have an important effect on the performance of tasks typically used to assess short-term memory in monkeys, such as matching-to-sample or delayed-response tasks, especially if monkeys use selective attention as a strategy for solving the problems posed by such tasks (see di Pellegrino and Wise 1993b; Awh and Jonides 2001).

Although attention could account for many findings about PF, we do not aim to replace one monolithic theory of PF function—the maintenance-memory theory—with an equally monolithic “attention theory.” Delay-period activity appears to reflect the learning and implementation of behavior-guiding rules (Wise et al. 1996b; White and Wise 1999; Wallis et al. 2001, Wallis and Miller 2003), categorization of events and stimuli (Freedman et al. 2001, 2003), prediction of forthcoming events (Rainer et al. 1999), task selection (Hoshi et al. 1998; Asaad et al. 2000), and adaptive actions within structured-event sequences (Barone and Joseph 1989; Quintana and Fuster 1999; Ninokura et al. 2003, 2004; Hoshi and Tanji 2004), among other cognitive functions. According to one view, PF functions in general intelligence for the solution of any and all difficult cognitive problems (Duncan and Owen 2000). Gaffan (2002) has likewise argued that PF resembles a global workspace, in the sense used by Baars et al. (2003), implying a lack of domain selectivity. The present result, by showing that PFdl's delay-period activity lacks an account solely in terms of maintenance memory, supports these ideas to some extent. However, the finding of regional specializations among different parts of the PFdl (see Figure 4), in accord with similar findings (Ninokura et al. 2003, 2004; Hoshi and Tanji 2004), suggests that various parts of PF contribute to this global workspace differently, each by making some selective contribution to PF's overall function. Taken together, these observations suggest that delay-period activity in PF reflects functions extending far beyond maintenance memory to include all of the behaviors important to the life of primates.

## Materials and Methods

### 

#### Behavioral task, apparatus, and single-unit recordings

We trained two rhesus monkeys *(Macaca mulatta)* to perform the task. Each monkey sat in a primate chair in front of a computer monitor placed 57 cm from the monkey's eyes. We recorded eye position with an infrared oculometer and sampled at 250 Hz. The monkeys pressed a waist-high button with their right hand to start each trial and did not release the button until the end of the trial. Once the monkeys pressed the button, a 0.2° fixation point appeared at the center of the screen. After they had fixated this stimulus for 1.0–1.5 s, a 2° solid, gray circle appeared 8° from the center of the screen in one of four places. Figure 1A, part a illustrates the right (0°) location. After another 1.0–1.5 s, the circle revolved from this initial location to one of four final places (Figure 1A, part b) at 90°/s along a circular trajectory centered on the fixation point. For monkey 1, the circle revolved 90° or 180° either clockwise or counterclockwise. For monkey 2, the circle revolved 90°, 180°, or 270° either clockwise or counterclockwise. After the circle stopped, a 1.0 to 2.5-s delay period ensued. Then a trigger signal occurred, which provided an instruction as to the saccade target, as well as a “go” cue for the saccade. The trigger signal consisted of a 150-ms-long change in the circle's brightness (Figure 1A, part c), followed by its disappearance (Figure 1A, part d). If the circle dimmed, the saccade had to be directed to the circle's final (and current) location on that trial; if the circle brightened, the saccade had to be directed to the circle's initial location on that trial. After the monkeys started a saccade, the central fixation spot disappeared. If the monkeys made a saccade to the correct location, a new 0.2° fixation spot appeared there, and the monkeys had to fixate this spot for 1.0–1.5 s, after which it dimmed. The monkeys could then release the button to produce a fruit juice reward. If the monkeys broke fixation prior to the trigger signal, made an incorrect saccade, or released the button prematurely, the trial was cancelled, and the monkeys could begin a new trial. In control trials, the circle either did not move (both monkeys) or returned to its initial location (360° movement, either clockwise or counterclockwise, monkey 2 only), and the monkeys had to make a saccade to the location of the circle whether it dimmed or brightened. The initial and final locations of the circle and whether it brightened or dimmed were selected pseudorandomly, as was the duration of the delay period and the direction in which the circle revolved around the central fixation point. The monkeys had to complete one correct trial of each type (32 in all, including control trials) before repeating a trial type.

After the monkeys learned the task, we implanted recording chambers over the left (monkeys 1 and 2) and right (monkey 1) PFdl. For monkey 1, we used a single-electrode microdrive to obtain single-neuron activity records; for monkey 2, we used a microdrive that independently moved up to seven electrodes. During recordings in monkey 1, we intentionally biased the selection of task-related neurons toward those with delay-period activity. In monkey 2, we recorded the activity of all isolated neurons, regardless of whether they were task related.

For histological reconstruction of recording sites, we examined Nissl-stained sections of 40 μm thickness from the right hemisphere in monkey 1 and the left hemisphere in monkey 2.

#### Quantification of tuning

We represented firing rate data in a 4 × 4 matrix, *F_ij_,* with rows *(i)* corresponding to the remembered location and columns *(j)* to the attended location (Figures 2, 3A–3C, S1–S3, S5, and S6). We assessed tuning for the remembered locations by comparing the variability of firing rate between trials in different rows with the variability of firing rate between trials from the same row. To avoid the influence of across-column modulations (i.e., an attention effect), both between-row and within-row variabilities were calculated only for matrix elements from the same column, one column at a time, and then we averaged these results. This procedure amounts to comparing different remembered locations, while holding the attended location fixed. To quantify the strength of tuning for the remembered location, we computed a ratio of between-row variability and within-trial type variability:



(1)

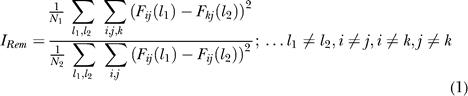



where *l_1_* and *l_2_* index individual trials, *i, j,* and *k* are matrix indexes that take on the values of 0°, 90°, 180°, and 270°, *F_ij_ (l)* is the firing rate on the *l^th^* trial for which position *i* was the remembered location and position *j* was the attended location, and *N_1_* and *N_2_* are total number of elements in the respective sums. Control trials were excluded from the calculation by not considering the diagonal elements of *F_ij_* (i = j, j = k).

We evaluated tuning to the attended location similarly by comparing across-column variability with within-column variability, one row at a time. The strength of representation of the attended location, was quantified as:



(2)

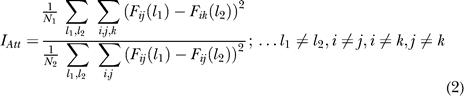



We used two task periods to compute the single trial firing rates *F_ij_ (l).* To classify neurons into those representing remembered versus attended location, we used an 800-ms period preceding the trigger signal. We also evaluated spatial tuning *(I)* during the final 800-ms period before the circle started to move. (Figures S5A and S6A illustrate this “early” period slightly differently, averaging activity in an interval from 200 ms to 1,000 ms after the appearance of the circle.) The *I_Rem_* or *I_Att_* ratios approximated unity for untuned neurons and increased with tuning strength. To measure statistical significance, we used a randomization test. Trials were randomly shuffled among different remembered or attended locations, and the indexes were recomputed. This procedure was repeated 1,000 times to yield a distribution of index values from which we computed a probability *p.* We chose a statistical significance level of *p < 0.01* to classify neurons as tuned for either *I_Rem_, I_Att_,* both, or neither (untuned).

#### Population histograms

We computed the population histograms of Figure 6 by first determining each neuron's preferred location, using firing rates during the 800 ms preceding the trigger signal. Then we ranked the trials as belonging to the preferred location, the next most preferred, the third most preferred, and the least preferred location. This ranking was then applied to the other task periods. Next we calculated peri-event time histograms (PETHs) for each rank, separately for each neuron. Averages of these single-neuron PETHs yielded the population histograms. To avoid biasing average histograms by statistical noise in the ranks, we used one half of the trials to compute the ranks and the other half to compute the histograms. If the spatial preference of a neuron merely reflected noise, this procedure tended to nullify the influence of the neuron on the population average. We ranked attended and remembered locations in separate computations.

#### Neuron-dropping curves

Neuron-dropping curves (Figures 7A–7D and S7) estimated how well ensembles of PF neurons represented the remembered and attended locations of the circle (Wessberg et al. 2000). We excluded control trials, in which the circle either did not move or moved 360°, from this analysis. The method measured the probability that the attended and remembered locations could be correctly estimated using a single trial of activity from a neuronal ensemble as a function of its size. The calculation started with a random selection of *n* neurons from a population. Then, for a given condition (e.g., a remembered location *i* of 0° and an attended location *j* of 90°), we selected one trial of that condition randomly from each neuron (test trials). All the other trials for that neuron contributed to a look-up table of firing rates. This look-up table consisted of a matrix of average firing rates *<F_ij_>* for remembered locations, *i,* and attended locations, *j.* The differences between firing rates in the look-up table and the rate on the selected trial were rank ordered, with a smaller rank signifying a closer match. We then summed the ranks *r_ij_* across individual neurons and took the remembered and attended locations associated with the lowest combined rank as the population estimation. The estimated remembered location either agreed or disagreed with the actual remembered location of the selected trial, as did the estimated attended location in a separate computation. Repeating this procedure for a given number of neurons, *n,* more than 2,400 times—each time starting with a randomly selected set of test trials (more than 200 trials from each of the 12 conditions; four controls excluded)—yielded a percentage of correct estimations of the attended and remembered locations. We then calculated neuron-dropping curves for ensembles of size one to the total number of neurons, but typically the range 1–100 sufficed to capture the main features of the population estimation.

To assess the representation of attended and remembered locations during the delays (see Figure 7A–7D), we calculated neuron-dropping curves for the 800-ms period immediately preceding the onset of circle movement (gray curves in Figure 7A–7D) and the 800-ms period immediately preceding the trigger signal (colored curves). Finally, we evaluated the time course of changes in these estimations, using neuron-dropping curves for a 200-ms window, which moved in 50-ms steps along the trigger-aligned records (Figures 7E, 7F, and 8). The 200-ms window measured activity immediately before the time point plotted, to prevent the artifactual early appearance of a signal detection, and thus represents a “backward-looking” average.

## Supporting Information

Figure S1 Rasters and Histograms from a Representative Attention Cell The activity matrix is the same as in Figure 3A, measured in the 800 ms immediately prior to the trigger signal. This neuron is not the same as that illustrated in Figure 2. Beneath the activity matrix, the rasters and histograms for each attended and remembered location are displayed in the format of Figure 2A.
(103 KB PPT).

Figure S2 Rasters and Histograms from a Representative Memory Cell The activity matrix is the same as in Figure 3B, measured in the 800 ms prior to the trigger stimulus. Format as in Figure S1.
(95 KB PPT).

Figure S3 Rasters and Histograms from Two Representative Hybrid Cells The activity matrix in (A) is the same as in Figure 3C, part a; the one in (B) is the same as in Figure 3C, part b. Format as in Figure S1.
(151 KB PPT).

Figure S4 Activity Early Versus Late in the Delay Period A table of tuning indexes is given at the top for each of the cell classes (plotted in the bottom part of the figure), combinations of those classes, and other groups of cells as described in the left column. These population averages are divided into two groups of columns, those on the left showing data for the period before the circle began rotating (early) and those on the right showing data for the period after it had stopped and the monkey awaited the trigger signal (late). In the plot, the dashed line shows the median values, the dotted line shows the upper IQR.
(56 KB PPT).

Figure S5 Activity Early Versus Late in the Delay Period Same PFdl neuron as in Figure 2. The activity matrix in (C) comes from the data in (A), and the matrix in (D) comes from the data in (B), in the format of Figure 2C. In (A), the red boxes enclose the measured period for the preferred location, 800 ms prior to the beginning of the circle's movement (200–1,000 ms after circle onset). In (D), the box shows the 800 ms immediately prior to the trigger stimulus. Note that the column-to-column variation in C necessarily results from chance variation because at that time the circle's final location is unknown. The figure shows, by example, that the spatial tuning in the period just before the triggering event strongly exceeds that before the circle begins moving, thus ruling out a strictly sensory account for spatial tuning (see also Figure S4). Note that after circle movement, responses to the circle were greater at the cell's preferred location (90°) but smaller at the least preferred location (270°).
(188 KB PPT).

Figure S6 Activity Early Versus Late in the Delay Period Same PFdl neuron as in Figure S1, in the format of Figure S5. The red boxes show the measured period for the cell's preferred location in both (A) and (B).
(156 KB PPT).

Figure S7 Neuron-Dropping Curves for the Two Monkeys Combined Format as in Figure 7A–7D.
(46 KB PPT).

## Abbreviations

Att-trial: attended-location trial
*I_Att_*: attended-location index
IQR: interquartile range
*I_Rem_*: remembered-location index
PETH: peri-event time histogram
PF: prefrontal cortex
PFdl: dorsolateral prefrontal cortex
Rem-trial: remembered-location trial
