# Supplementary material for: Representation of Attended Versus Remembered Locations in Prefrontal Cortex
Source: PLoS Biol. 2004 Oct 26;2(11):e365. doi: 10.1371/journal.pbio.0020365 (PMC524249; doi:10.1371/journal.pbio.0020365)
Supplement: Figure S1 — The activity matrix is the same as in Figure 3A, measured in the 800 ms immediately prior to the trigger signal. This neuron is not the same as that illustrated in Figure 2. Beneath the activity matrix, the rasters and histograms for each attended and remembered location are displayed in the format of Figure 2A. (103 KB PPT). [file pbio.0020365.sg001.ppt]

## Slide 1
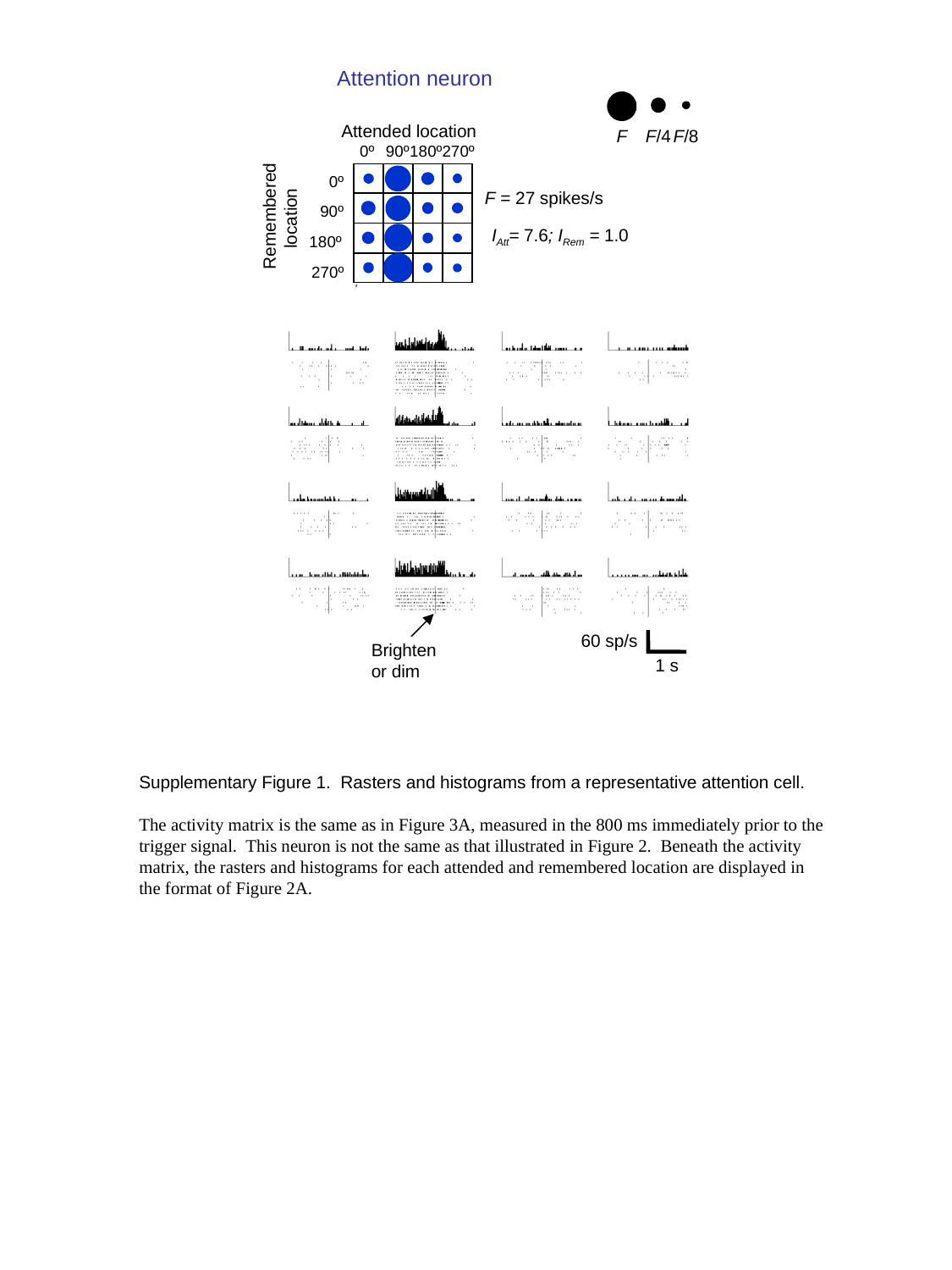

Attention neuron
Attended location
F
F/4
F/8
0º
90º
180º
270º
0º
F = 27 spikes/s
Remembered
location
90º
IAtt= 7.6; IRem = 1.0
180º
270º
60 sp/s
Brighten
or dim
1 s
Supplementary Figure 1. Rasters and histograms from a representative attention cell.
The activity matrix is the same as in Figure 3A, measured in the 800 ms immediately prior to the trigger signal. This neuron is not the same as that illustrated in Figure 2. Beneath the activity matrix, the rasters and histograms for each attended and remembered location are displayed in the format of Figure 2A.
