# Supplementary material for: Representation of Attended Versus Remembered Locations in Prefrontal Cortex
Source: PLoS Biol. 2004 Oct 26;2(11):e365. doi: 10.1371/journal.pbio.0020365 (PMC524249; doi:10.1371/journal.pbio.0020365)
Supplement: Figure S2 — The activity matrix is the same as in Figure 3B, measured in the 800 ms prior to the trigger stimulus. Format as in Figure S1. (95 KB PPT). [file pbio.0020365.sg002.ppt]

## Slide 1
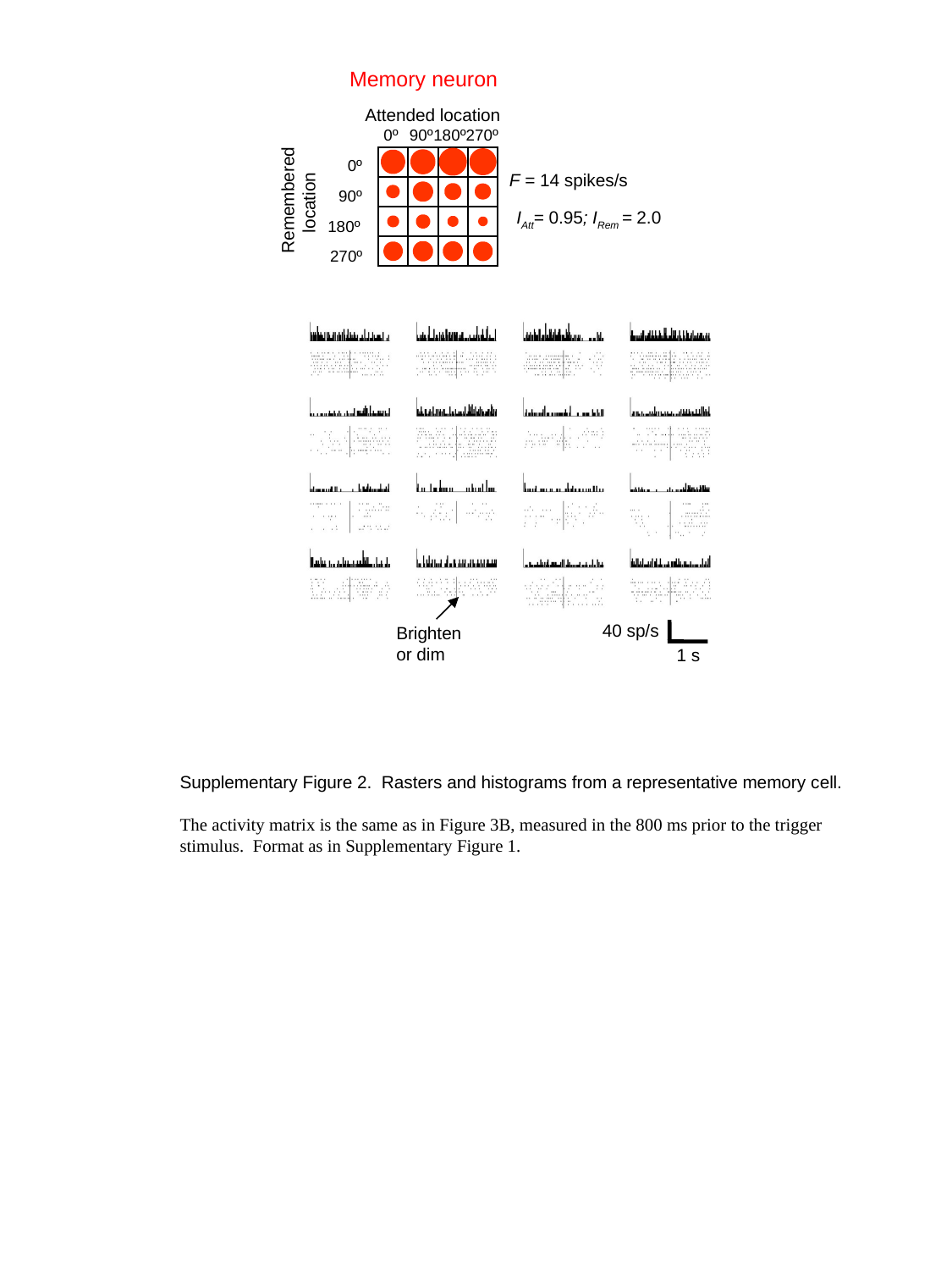

Memory neuron
Attended location
0º
90º
180º
270º
0º
F = 14 spikes/s
Remembered
location
90º
IAtt= 0.95; IRem = 2.0
180º
270º
40 sp/s
Brighten
or dim
1 s
Supplementary Figure 2. Rasters and histograms from a representative memory cell.
The activity matrix is the same as in Figure 3B, measured in the 800 ms prior to the trigger stimulus. Format as in Supplementary Figure 1.
