# Supplementary material for: Representation of Attended Versus Remembered Locations in Prefrontal Cortex
Source: PLoS Biol. 2004 Oct 26;2(11):e365. doi: 10.1371/journal.pbio.0020365 (PMC524249; doi:10.1371/journal.pbio.0020365)
Supplement: Figure S3 — The activity matrix in (A) is the same as in Figure 3C, part a; the one in (B) is the same as in Figure 3C, part b. Format as in Figure S1. (151 KB PPT). [file pbio.0020365.sg003.ppt]

## Slide 1
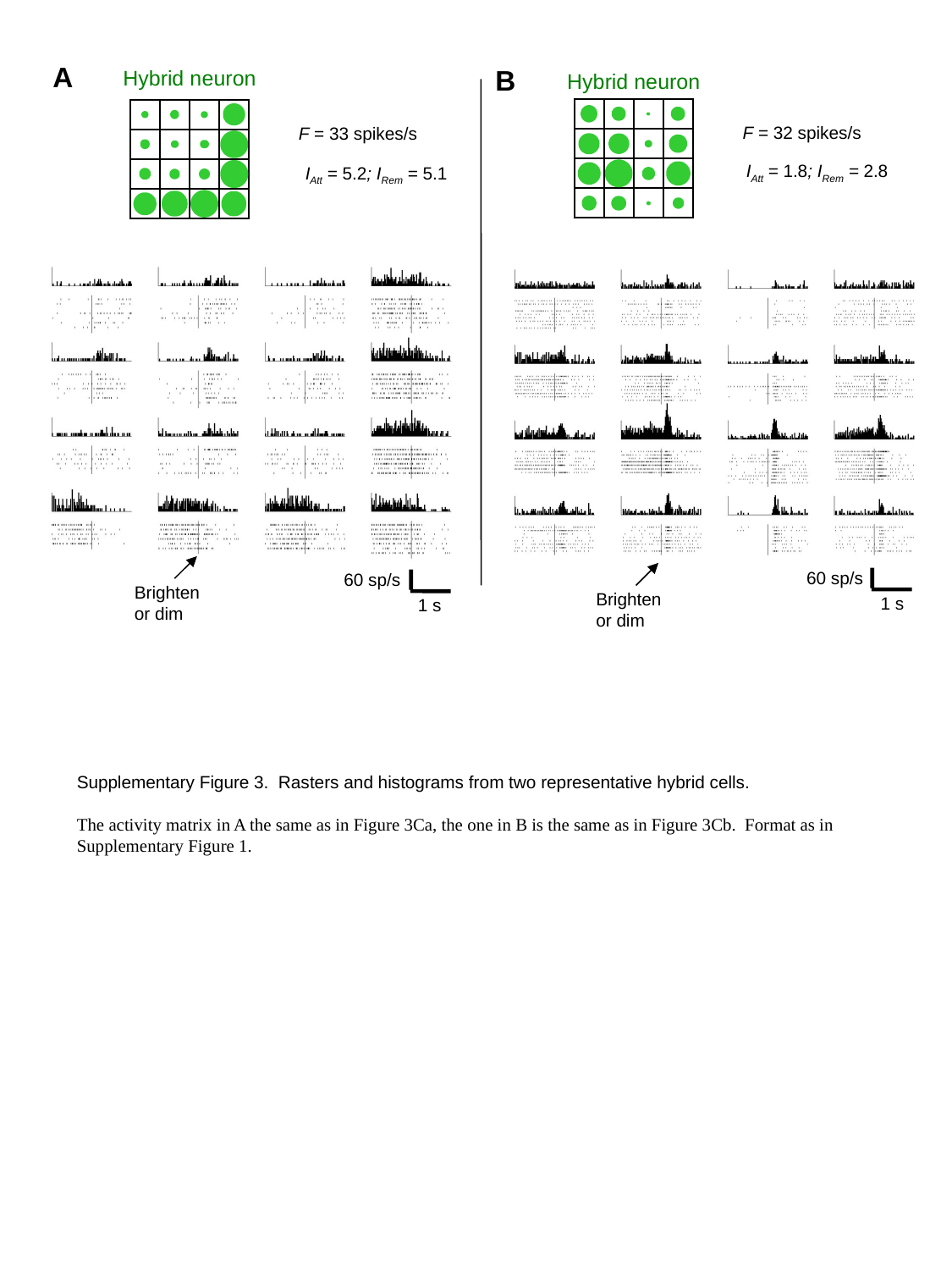

A
B
Hybrid neuron
Hybrid neuron
F = 32 spikes/s
IAtt = 1.8; IRem = 2.8
F = 33 spikes/s
IAtt = 5.2; IRem = 5.1
60 sp/s
60 sp/s
Brighten
or dim
Brighten
or dim
1 s
1 s
Supplementary Figure 3. Rasters and histograms from two representative hybrid cells.
The activity matrix in A the same as in Figure 3Ca, the one in B is the same as in Figure 3Cb. Format as in Supplementary Figure 1.
