# Supplementary material for: Representation of Attended Versus Remembered Locations in Prefrontal Cortex
Source: PLoS Biol. 2004 Oct 26;2(11):e365. doi: 10.1371/journal.pbio.0020365 (PMC524249; doi:10.1371/journal.pbio.0020365)
Supplement: Figure S4 — A table of tuning indexes is given at the top for each of the cell classes (plotted in the bottom part of the figure), combinations of those classes, and other groups of cells as described in the left column. These population averages are divided into two groups of columns, those on the left showing data for the period before the circle began rotating (early) and those on the right showing data for the period after it had stopped and the monkey awaited the trigger signal (late). In the plot, the dashed line shows the median values, the dotted line shows the upper IQR. (56 KB PPT). [file pbio.0020365.sg004.ppt]

## Slide 1
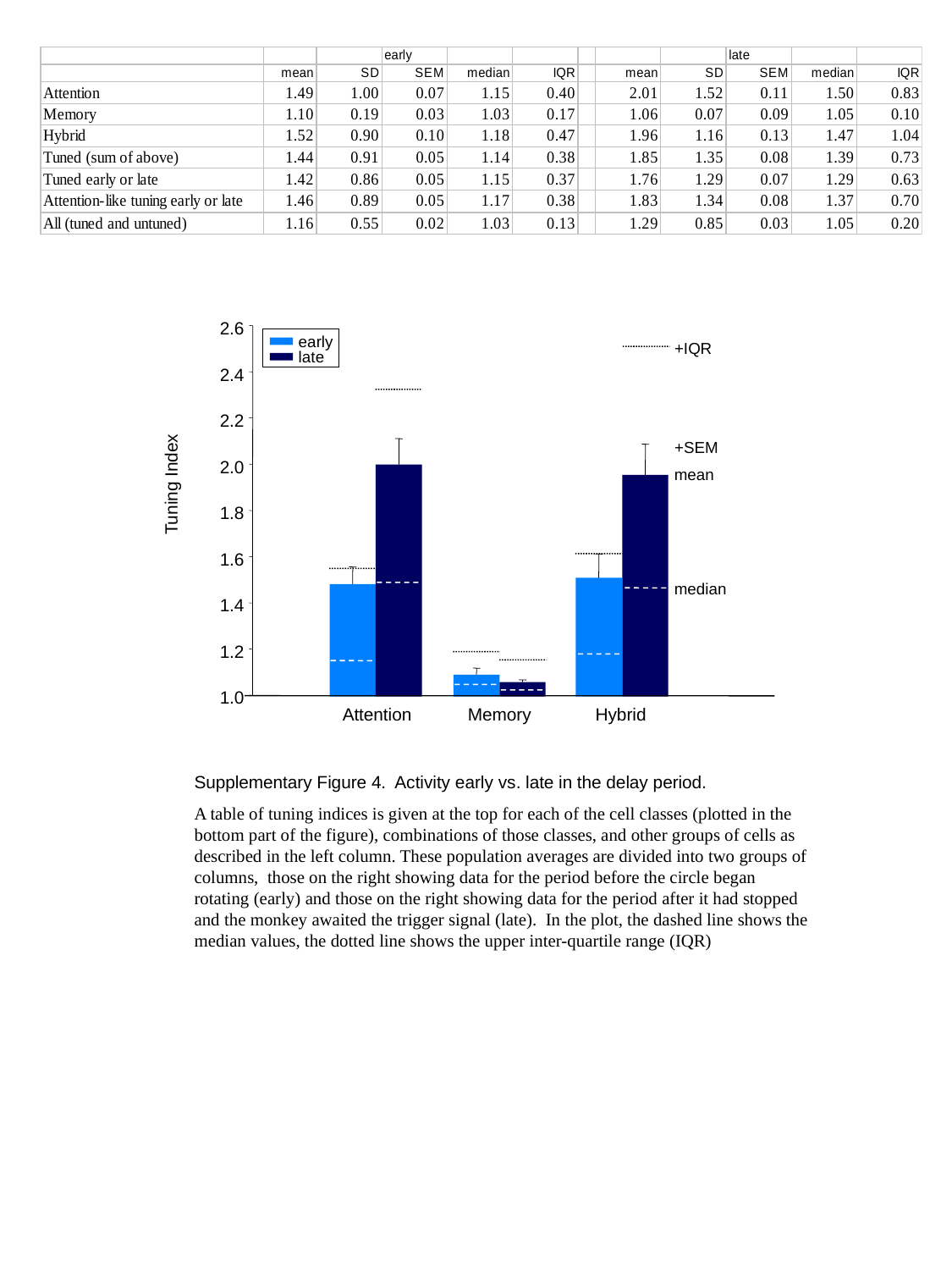

2.6
early
+IQR
late
2.4
2.2
+SEM
2.0
mean
Tuning Index
1.8
1.6
median
1.4
1.2
1.0
Attention
Memory
Hybrid
Supplementary Figure 4. Activity early vs. late in the delay period.
A table of tuning indices is given at the top for each of the cell classes (plotted in the bottom part of the figure), combinations of those classes, and other groups of cells as described in the left column. These population averages are divided into two groups of columns, those on the right showing data for the period before the circle began rotating (early) and those on the right showing data for the period after it had stopped and the monkey awaited the trigger signal (late). In the plot, the dashed line shows the median values, the dotted line shows the upper inter-quartile range (IQR)
