# Supplementary material for: Representation of Attended Versus Remembered Locations in Prefrontal Cortex
Source: PLoS Biol. 2004 Oct 26;2(11):e365. doi: 10.1371/journal.pbio.0020365 (PMC524249; doi:10.1371/journal.pbio.0020365)
Supplement: Figure S5 — Same PFdl neuron as in Figure 2. The activity matrix in (C) comes from the data in (A), and the matrix in (D) comes from the data in (B), in the format of Figure 2C. In (A), the red boxes enclose the measured period for the preferred location, 800 ms prior to the beginning of the circle's movement (200–1,000 ms after circle onset). In (D), the box shows the 800 ms immediately prior to the trigger stimulus. Note that the column-to-column variation in C necessarily results from chance variation because at that time the circle's final location is unknown. The figure shows, by example, that the spatial tuning in the period just before the triggering event strongly exceeds that before the circle begins moving, thus ruling out a strictly sensory account for spatial tuning (see also Figure S4). Note that after circle movement, responses to the circle were greater at the cell's preferred location (90°) but smaller at the least preferred location (270°). (188 KB PPT). [file pbio.0020365.sg005.ppt]

## Slide 1
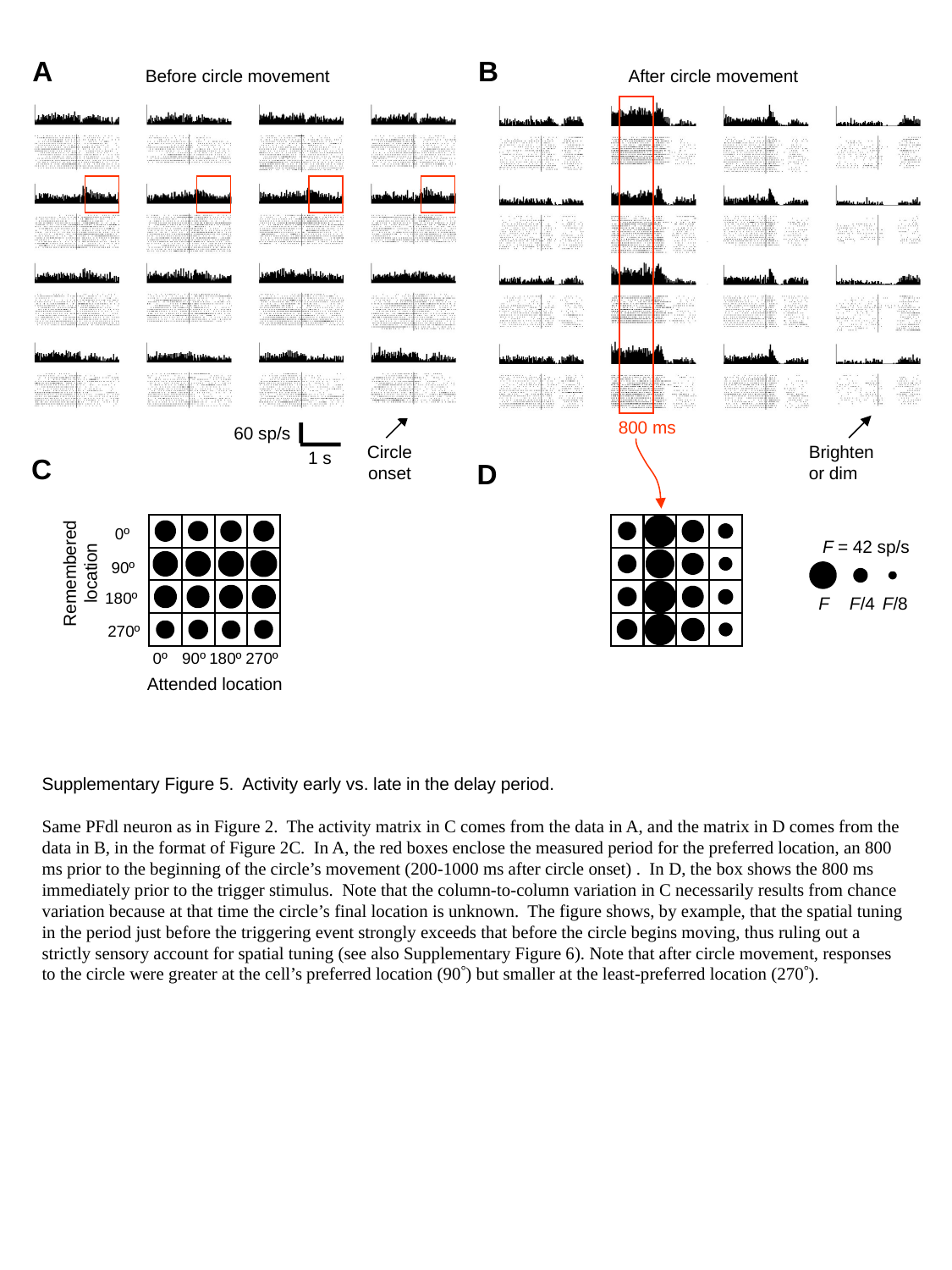

B
A
Before circle movement
After circle movement




800 ms
60 sp/s
Circle
onset
Brighten
or dim
1 s
C
D
0º
F = 42 sp/s
F
F/4
F/8
Remembered
 location
90º
180º
270º
0º
90º
180º
270º
Attended location
Supplementary Figure 5. Activity early vs. late in the delay period.
Same PFdl neuron as in Figure 2. The activity matrix in C comes from the data in A, and the matrix in D comes from the data in B, in the format of Figure 2C. In A, the red boxes enclose the measured period for the preferred location, an 800 ms prior to the beginning of the circle’s movement (200-1000 ms after circle onset) . In D, the box shows the 800 ms immediately prior to the trigger stimulus. Note that the column-to-column variation in C necessarily results from chance variation because at that time the circle’s final location is unknown. The figure shows, by example, that the spatial tuning in the period just before the triggering event strongly exceeds that before the circle begins moving, thus ruling out a strictly sensory account for spatial tuning (see also Supplementary Figure 6). Note that after circle movement, responses to the circle were greater at the cell’s preferred location (90°) but smaller at the least-preferred location (270°).
