# Supplementary material for: Representation of Attended Versus Remembered Locations in Prefrontal Cortex
Source: PLoS Biol. 2004 Oct 26;2(11):e365. doi: 10.1371/journal.pbio.0020365 (PMC524249; doi:10.1371/journal.pbio.0020365)
Supplement: Figure S6 — Same PFdl neuron as in Figure S1, in the format of Figure S5. The red boxes show the measured period for the cell's preferred location in both (A) and (B). (156 KB PPT). [file pbio.0020365.sg006.ppt]

## Slide 1
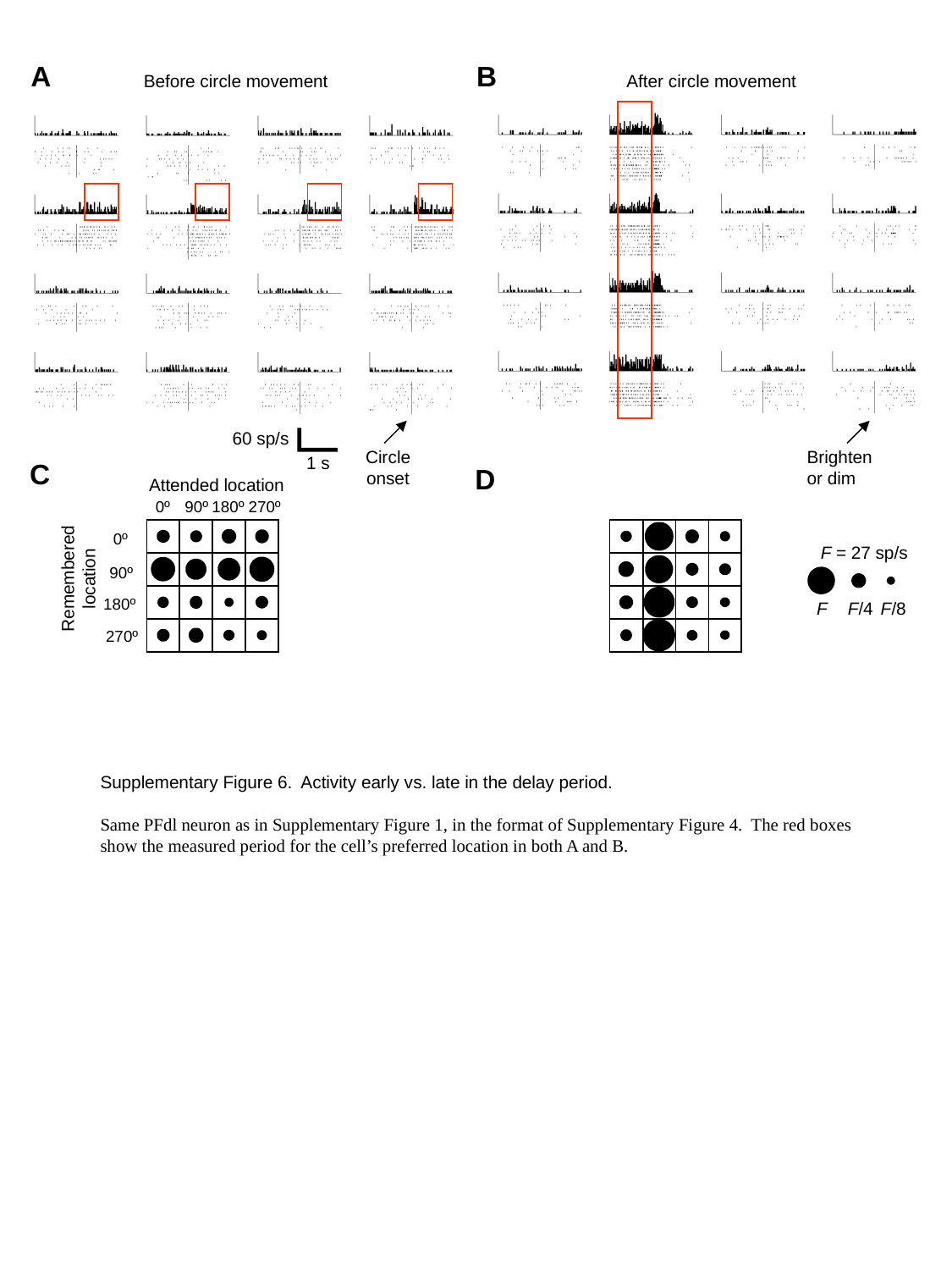

B
A
Before circle movement
After circle movement
60 sp/s
Circle
onset
Brighten
or dim
1 s
C
D
Attended location
0º
90º
180º
270º
0º
F = 27 sp/s
F
F/4
F/8
Remembered
 location
90º
180º
270º
Supplementary Figure 6. Activity early vs. late in the delay period.
Same PFdl neuron as in Supplementary Figure 1, in the format of Supplementary Figure 4. The red boxes show the measured period for the cell’s preferred location in both A and B.
