# Supplementary material for: Representation of Attended Versus Remembered Locations in Prefrontal Cortex
Source: PLoS Biol. 2004 Oct 26;2(11):e365. doi: 10.1371/journal.pbio.0020365 (PMC524249; doi:10.1371/journal.pbio.0020365)
Supplement: Figure S7 — Format as in Figure 7A–7D. (46 KB PPT). [file pbio.0020365.sg007.ppt]

## Slide 1
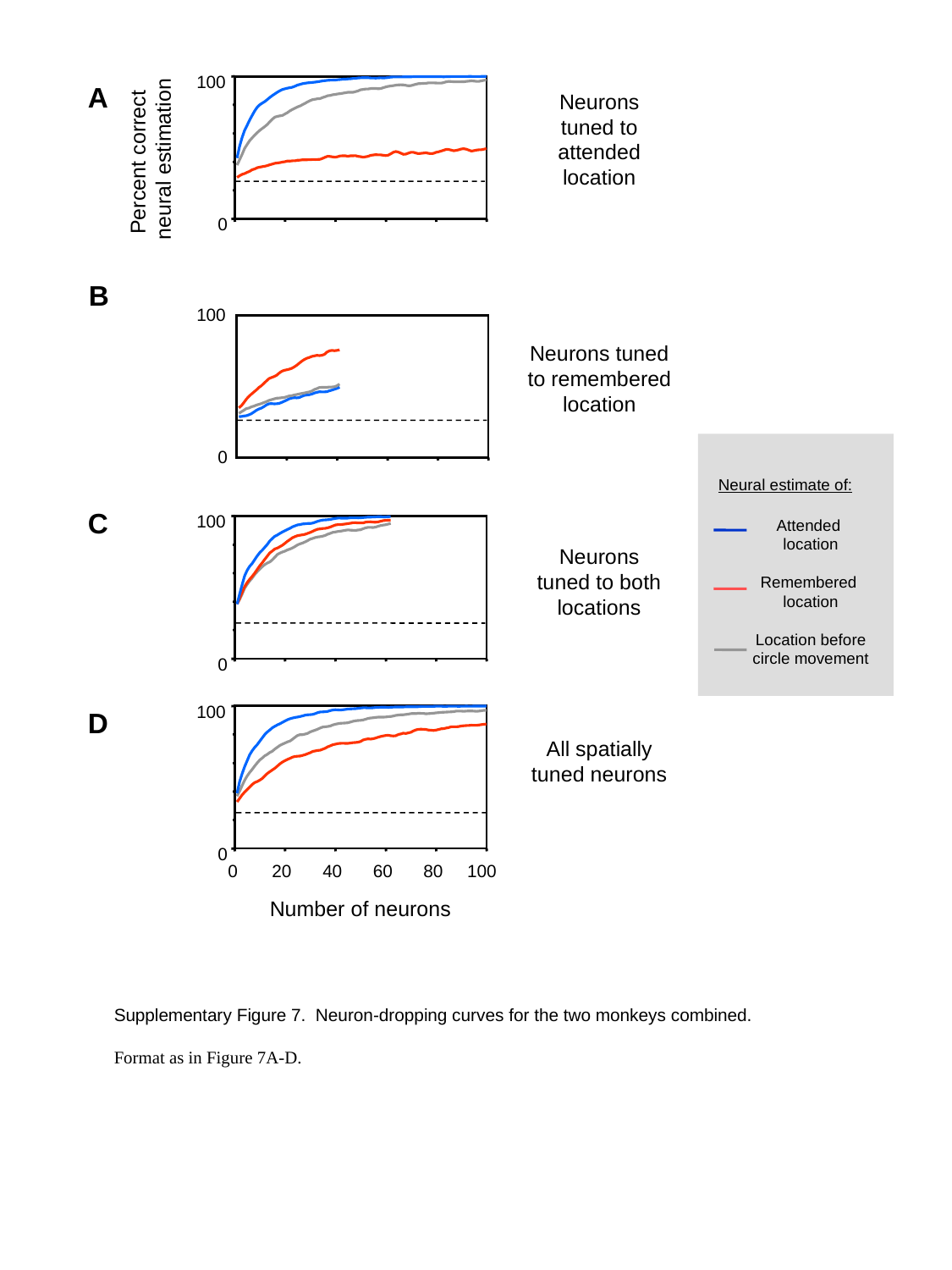

100
A
Neurons tuned to attended location
Percent correct
 neural estimation
0
B
100
Neurons tuned to remembered location
0
Neural estimate of:
Attended
location
Remembered
location
Location before
circle movement
C
100
Neurons tuned to both locations
0
D
100
All spatially tuned neurons
0
0
20
40
60
80
100
Number of neurons
Supplementary Figure 7. Neuron-dropping curves for the two monkeys combined.
Format as in Figure 7A-D.
